# Honokiol improves depression-like behaviors in rats by HIF-1α- VEGF signaling pathway activation

**DOI:** 10.3389/fphar.2022.968124

**Published:** 2022-08-25

**Authors:** Xiao-Xu Fan, Wen-Yan Sun, Yu Li, Qin Tang, Li-Na Li, Xue Yu, Shu-Yan Wang, Ang-Ran Fan, Xiang-Qing Xu, Hong-Sheng Chang

**Affiliations:** ^1^ School of Chinese Materia Medica, Beijing University of Chinese Medicine, Beijing, China; ^2^ School of Traditional Chinese Medicine, Beijing University of Chinese Medicine, Beijing, China; ^3^ Experiment Center, Encephalopathy Department, Affiliated Hospital of Shandong University of Chinese Medicine, Jinan, China

**Keywords:** honokiol, antidepressant effect, HIF-1α-VEGF signaling pathway, synapse plasticity, molecular docking

## Abstract

Increasing evidence indicates that the pathogenesis of depression is closely linked to impairments in neuronal synaptic plasticity. Honokiol, a biologically active substance extracted from *Magnolia Officinalis*, has been proven to exert significant antidepressant effects. However, the specific mechanism of action remains unclear. In this study, PC12 cells and chronic unpredictable mild stress (CUMS) model rats were used to explore the antidepressant effects and potential mechanisms of honokiol *in vitro* and in rats. *In vitro* experiment, a cell viability detection kit was used to screen the concentration and time of honokiol administration. PC12 cells were administered with hypoxia-inducible factor-1α (HIF-1α) blocker, 2-methoxyestradiol (2-ME), and vascular endothelial growth factor receptor 2 (VEGFR-2) blocker, SU5416, to detect the expression of HIF-1α, VEGF, synaptic protein 1 (SYN 1), and postsynaptic density protein 95 (PSD 95) by western blotting. In effect, we investigated whether the synaptic plasticity action of honokiol was dependent on the HIF-1α-VEGF pathway. *In vivo*, behavioral tests were used to evaluate the reproducibility of the CUMS depression model and depression-like behaviors. Molecular biology techniques were used to examine mRNA and protein expression of the HIF-1α-VEGF signaling pathway and synaptic plasticity-related regulators. Additionally, molecular docking techniques were used to study the interaction between honokiol and target proteins, and predict their binding patterns and affinities. Experimental results showed that honokiol significantly reversed CUMS-induced depression-like behaviors. Mechanically, honokiol exerted a significant antidepressant effect by enhancing synaptic plasticity. At the molecular level, honokiol can activate the HIF-1α-VEGF signaling pathway *in vitro* and *in vivo*, as well as promote the protein expression levels of SYN 1 and PSD 95. Taken together, the results do not only provide an experimental basis for honokiol in the clinical treatment of depression but also suggest that the HIF-1α-VEGF pathway may be a potential target for the treatment of depression.

## Introduction

Depression is a severe psychiatric disorder characterized by a significant and persistent low mood (Niu and Snyder, 2022). Common symptoms include slow thinking, impaired cognitive function, diminished volitional activity, sleep disturbances, and appetite loss (LeMoult and Gotlib, 2019). With the development of society, the incidence rate of depression is increasing due to several factors, such as social, environmental, psychological, and genetic factors. Depression, one of the main causes of diseases globally, severely endangers the physical and mental health of individuals. No effective drug is available to treat depression, which exhibits a disturbingly complex pathogenesis. Most classical antidepressants currently used in clinical practice, such as tricyclic antidepressants (TCAs), monoamine oxidase inhibitors (MAOIs), and selective serotonin (5-HT) reuptake inhibitors (SSRIs), are only effective in 30%–40% of patients. Besides, they have drug resistance and, high recurrence rate. Long-term use can cause sleep disorders, gastrointestinal disorders, cardiotoxicity, sexual dysfunction, and other side effects (Racagni and Popoli, 2010; Correll et al., 2015; Blackburn, 2019). Therefore, due to the urgent need to discover safer and more effective drugs for treating depression, in-depth research on the pathogenesis of depression is becoming increasingly important.

Honokiol is a polyphenol compound with few toxic side effects and several pharmacological effects. In recent years, honokiol has been found to have an obvious protective effect on the nervous system by regulating neurotrophic factors and promoting nerve regeneration (Woodbury et al., 2013; Talarek et al., 2017). Additionally, it has other pharmacological effects, such as free radical scavenging, anti-inflammatory, analgesic, antioxidant, antibacterial, and antitumor effects (Wang et al., 2020; Chen et al., 2021; Trifan et al., 2021). Honokiol has potent antidepressant activity when it is used alone or in combintion. Studies have shown that honokiol combined with ginger oil has an antidepressant effect in CUMS model rats (Qiang et al., 2009). The alcohol extract of Banxia Houpu Decoction is rich in honokiol, which can reduce the changes in brain neurotransmitters and prevent immune and inflammatory reactions to exert antidepressant properties on rodent depression models (Wang et al., 2005). Honokiol has protective effects on brain regions, such as the hippocampus and cortical neurons (Borgonetti et al., 2021). Furthermore, it can block hippocampal endoplasmic reticulum stress to eliminate cognitive impairment and depression-like behaviors induced by chronic restraint stress (Jangra et al., 2016). Other studies have reported that honokiol normalized the function of the hypothalamus-pituitary-adrenal (HPA) axis and increased the level of brain-derived neurotrophic factor in the hippocampus, which has an antidepressant effect on CUMS rat models (Wang et al., 2018). In addition to the above studies, numerous experimental studies have shown that honokiol has therapeutic effects in acute and chronic stress models, lipopolysaccharide depression model, and corticosterone-induced depression model (Xu et al., 2008; Pitta et al., 2013; Sulakhiya et al., 2014). Honokiol is a small molecule that readily enters the central nervous system through the blood-brain barrier to have a direct effect on nerve cells (Lin et al., 2012). It has wide potential to develop into a drug for neurological diseases. Therefore, clarifying the antidepressant mechanism of honokiol has a reference role in the development of novel antidepressants.

Hypoxia-inducible factor-1 (HIF-1) is a transcription factor of hypoxia response. As a major regulator of cell oxygen homeostasis in mammalian cells, HIF-1 is composed of the active subunit, HIF-1α, and the constitutive subunit, HIF-1β (Kaelin and Ratcliffe, 2008). Increasing the level of hypoxia-inducible factors may become a potential target for depression therapy (Kang et al., 2021). Research has proven that hypoxic preconditioning (HP) produces significant antidepressant effects by increasing the expression of HIF-1α in the hippocampus, hypothalamic paraventricular nucleus, and neocortex of rats (Baranova et al., 2010). The target genes of HIF-1, erythropoietin (EPO), and vascular endothelial growth factor (VEGF), have been proven to elicit antidepressant effects in animal models (Deyama et al., 2019). Indeed, VEGF plays an essential role in the antidepressant effects. Peripheral administration of EPO produces a robust antidepressant-like effect (Girgenti et al., 2009). More importantly, studies have found that activating the HIF-1α-VEGF signaling pathway can effectively reverse CUMS depression-like behaviors and memory impairment and promote synaptic growth in primary hippocampal neurons. The HIF-1α-VEGF pathway plays an important role in promoting hippocampal neurogenesis and synaptic plasticity *in vivo* and *in vitro* (Li G. et al., 2020). Collectively, activation of the HIF-1α-VEGF signaling pathway is a promising strategy to improve depression-like behaviors.

Our previous study found that 10 mg/kg of honokiol can significantly improve depression-like behaviors in acute and chronic stress mouse models (Wang et al., 2017). The aim of the present study was to investigate the necessity of the HIF-1α-VEGF pathway in enhancing neuronal synaptic plasticity with honokiol and to clarify the antidepressant effect of honokiol on CUMS depression rat models by activating the HIF-1α-VEGF pathway.

## Materials and methods

### Animals and cells

Male Sprague Dawley rats, specific pathogen-free (SPF) grade, weighing 200–220 g, were obtained from SiPeiFu Biotechnology Co., Ltd., [Animal license number: SYXK (Beijing) 2020-0033]. All animal procedures were approved by the Experimental Animal Ethics Committee of the Academic Committee of Beijing University of Chinese Medicine (Project identification code: BUCM-4-2021090301–3049). Rat pheochromocytoma PC12 cells were generously provided by the Research and Experiment Center, School of Traditional Chinese Medicine, Beijing University of Chinese Medicine.

### Drugs and reagents

**FIGURE 1:**
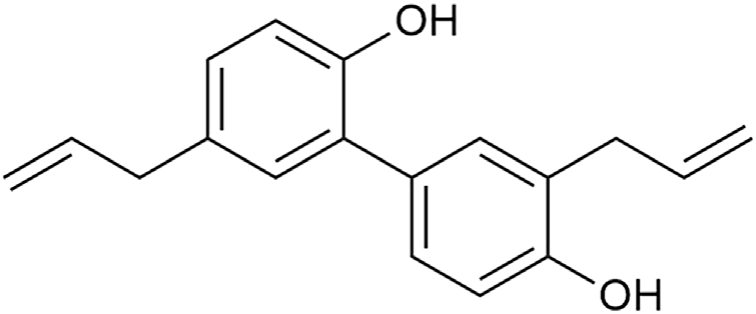
The structure of honokiol (Chemical formula: C_18_H_18_O_2_; molecular weight: 266.34).

Drugs used included paroxetine (catalog number: H13M10Z82647, Yuanye, Shanghai, China), SU5416 (catalog number: ab145056, Abcam, Cambridge, United States), 2-methoxyestradiol (catalog number: A4188, APE×BIO, Houston, United States), and honokiol (catalog number: SH8140, Solarbio, Beijing, China). The structure of honokiol is shown in Figure 1.

All reagents for cell culture were purchased from Gibco (Carlsbad, CA, United States). Protease inhibitor (catalog number: KGP603, Kaiji Biology, Jiangsu, China); phosphatase inhibitor (catalog number: KGP602, Kaiji Biology, Jiangsu, China). The primary antibodies and their dilution ratios in western blot were as follows: anti-HIF-1α (1:1,000, Abcam Cat# ab179483, RRID:AB_2732807), anti-phospho-PI3K (1:500, Abcam Cat# ab182651, RRID:AB_2756407), anti-VEGF (1:3000, Proteintech Cat#19003-1-AP, RRID:AB_2212657), anti-VEGFR-2 (1:800, Proteintech Cat# 26415-1-AP, RRID:AB_2756527), anti-PI3K (1:10000, Proteintech Cat# 60225-1-Ig, RRID:AB_11042594), anti-AKT (1:5,000, Proteintech Cat# 60203-2-Ig, RRID:AB_10912803), anti-phospho-AKT (1:5,000, Proteintech Cat# 66444-1-Ig, RRID:AB_2782958), anti-mTOR (1:25,000, Proteintech Cat# 66888-1-Ig, RRID:AB_2882219), anti-SYN 1 (1:6,000, Proteintech Cat# 20258-1-AP, RRID:AB_2800493), anti-PSD 95 (1:3,000, Proteintech Cat# 20665-1-AP, RRID:AB_2687961), anti-α-Tubulin (1:5,000, Proteintech Cat# 11224-1-AP, RRID:AB_2210206), anti-phospho-mTOR (1:1,000, Cell Signaling Technology Cat# 5536, RRID:AB_10691552). The secondary antibodies and their dilution ratios in western blot were as follows: HRP-conjugated affinipure goat anti-rabbit IgG (1:5,000, Proteintech Cat# SA00001-2, RRID: AB_2722564), and HRP-conjugated affinipure goat anti-mouse IgG (1:5,000, Proteintech Cat# SA00001-1, RRID: AB_2722565).

### Cell study design

#### Cell culture

**FIGURE 2:**
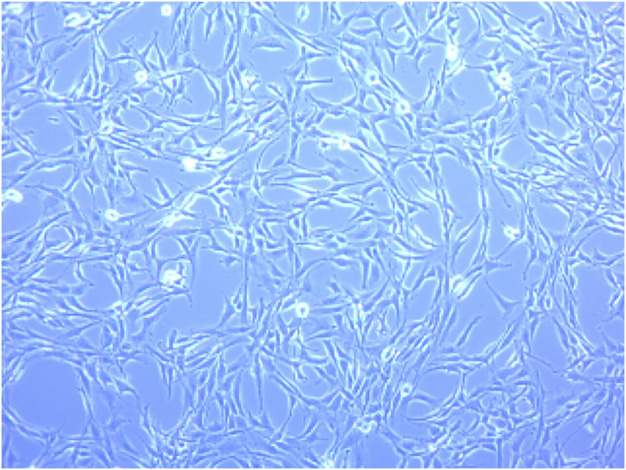
Morphology of normally cultured PC12 cells under the light microscope (×100).

PC12 cells were cultured in Dulbecco Modified Eagle Medium (DMEM; high glucose), which contained 10% fetal bovine serum and 1% penicillin/streptomycin at 37°C with 5% CO_2_. The medium was changed every 1 or 2 days. Generally, cell passage was performed when the cell fusion was approximately 80%. Cells in the logarithmic phase were used for subsequent experiments. Under suitable conditions, the cells adhered to the wall in the form of clusters with clearly visible edges. Their specific shape was shuttle-shaped with a strong refractive index, as shown in Figure 2.

#### Cell viability assay

The viability of PC12 cells was evaluated using Cell Counting Kit-8 (catalog number: CK04, Tongren, Japan). PC12 cells were seeded at a density of 5×10^3^ cells in one well of a 96-well plate. After culturing for 24 h, the cells were washed three times with 1 × PBS. Subsequently, PC12 cells were treated with 2, 5, 8, 10, and 16 μM honokiol at 37°C under 5% CO_2_ for 24 or 48 h. PC12 cells were incubated with CCK-8 working solution at 37°C for 1 h in the dark. Finally, the absorbance of each well was measured using a 96-well microplate reader (Shanghai Thermo Fisher Scientific, Inc.) at 450 nm. Relative cell viability was defined and calculated using the following formula: [A (experimental group)—A (blank)]/[A (control group)—A (blank)].

#### HIF-1α and VEGFR-2 blocking experiments

Cell experiments were divided into six groups: Control (Con), Con + Honokiol, Con + Honokiol + SU5416, Con + Honokiol + 2-ME, Con + SU5416, and Con + 2-ME groups. PC12 cells were cultured in 6-well plates at a density of 5×10^5^ cells per well for 24 h. After culturing, the cells were treated accordingly with honokiol (2 μM), 2-ME (5 μM), and SU5416 (4 μM). Specifically, cells in the Con + Honokiol group were treated with 2 μM honokiol for 24 h; cells in the Con + SU5416 group were pretreated with 4 μM SU5416 for 30 min and then replaced with new DMEM for 24 h; cells in the Con + Honokiol + SU5416 group were pretreated with 4 μM SU5416 for 30 min before honokiol treatment for 24 h; cells in the Con+2-ME group were pretreated with 5 μM 2-ME for 30 min and then replaced with new DMEM for 24 h; cells in the Con + Honokiol+2-ME group were pretreated with 5 μM 2-ME for 30 min before honokiol treatment for 24 h. After incubation, HIF-1α, VEGF, SYN 1, and PSD 95 protein expressions were detected by western blotting. SU5416, 2-ME, and honokiol were diluted in DMSO to corresponding concentrations, and the final concentration of DMSO was fixed at 0.1%. The dosages of these blockers have been previously reported by other studies (Fournier et al., 2012; Khan et al., 2015).

### Animal study design

#### Establishment of chronic unpredictable mild stress depression model

Before the experiment, all rats were fed normally for 7 days under standard laboratory conditions, including constant temperature (20–24°C), humidity (50–55%), standard ventilation system, and standard drinking water and food, to ensure that the rats adapt to the environment.

Rats were randomly divided into four groups (*n* = 8 in each group): Control (Con), CUMS (CUMS + saline), CUMS + Par (CUMS + 4.8 mg/kg paroxetine, i.p.), and CUMS + Honokiol (CUMS+10 mg/kg Honokiol, i.g.) groups. All rats, except those in the control group, were exposed to chronic unpredictable mild stress for 28 days. Stressors are shown in Table 1. The same stressor was not repeated within 7 days; otherwise, the rats would predict the stressors. Honokiol was suspended in 0.5% sodium carboxymethylcellulose (CMC-Na) at a certain concentration before use. The control and CUMS groups were given the same amount of saline. The CUMS + Par group received 4.8 mg/kg honokiol intraperitoneally. The CUMS + Honokiol group was given 10 mg/kg honokiol by intragastric administration. Each group of rats received the corresponding drug for 21 days after modeling. After the last dose on day 21, sucrose preference test and open field test were performed, respectively. After one of behavioral tests finished, rats were allowed to rest for a day before proceeding to the next one. The rats were weighed and anesthetized by intraperitoneal injection of 1% pentobarbital (2.75 ml/kg) before being sacrificed. In preparing the 1% pentobarbital, 0.9% saline was used. Finally, the hippocampus was taken for the detection of related indicators.

**TABLE 1:** Chronic unpredictable mild stress (CUMS) procedure.

Stressors	Details
Food and water deprivation	Rats were subjected to 24 h of food and water deprivation
Tail pinching	Tail pinch 1 cm from the beginning of the tail for 2 min
Physical restrain	Confinement in a tube for 2 h
Cold water swimming	Rats were placed in a cylindrical clear glass jar filled with 4°C cold water for 5 min
Wet bedding	Wet bedding for 24 h
Continuous illumination	Continuous illumination for 24 h
Cage tilt	45°cage inclined for 12 h

#### Open field test

The experiment was conducted in a quiet and stable light environment. Rats were brought to the testing room to adapt for 1 h before the OFT. The experimental setup consisted of two parts: the open field reaction chamber and the recording and analysis system. The open box with a circular bottom was 40 cm high, and 100 cm long in diameter. The bottom of the open box was divided equally into 25 small squares of equal areas. A digital camera was set up 2 m above the open field, which could cover the entire interior of the open field. A rat was placed in the center of an open box to adapt for 1 min, and then the small animal behavior recording analysis system (Etho-Vision XT9, Noldus, Netherlands) was used to record the distance and velocity of movement within 5 min. Before each rat was tested, the open box was cleaned with alcohol to remove all feces and odor left by previous rats.

#### Sucrose preference test

SPT was used to assess anhedonia in rats. Each rat was reared in a single cage during the test. The test consisted of two stages: 1) Adaptation stage: each rat was simultaneously given a bottle of 1% sucrose water and pure water for 48 h; 2) Test stage: after the adaptation phase, the rats were deprived of water for 24 h. Subsequently, each rat was given a bottle of 1% sucrose water and one bottle of pure water. Rats were free to drink during the test for 3 h. The consumption of sucrose water and pure water during the test was weighed. The sucrose preference was calculated as the percentage of sucrose consumption in the total consumption of sucrose water and pure water.

#### Quantitative real-time PCR assay

Total RNA was extracted from the hippocampi of rats using the Hipure Total RNA Mini Kit (catalog number: R4111-02, MAGEN). RNA concentration of every sample was measured using an ultraviolet spectrophotometer (UV-2000, Unico). Samples were placed on ice during the operation to prevent RNA degradation. The RevertAid First Strand cDNA Synthesis Kit (catalog number: K1622, Thermo Scientific) and the Power SYBR Green PCR Master Mix (catalog number: 4367659, Invitrogen) were used to reverse transcription. Thereafter, the following scheme was adopted: initial denaturation at 95°C for 10 min, denaturation at 95°C for 10 s, annealing at 60°C, and extension for 30 s. Fifty amplification cycles were completed. Amplification and quantification were performed using a Real-Time PCR device (Bio-Rad, USA). β-Actin was considered as the internal reference. The results were analyzed using the 2 ^−ΔΔCt^ method for relative quantification. The sequences of involved primers are shown in Table 2.

**TABLE 2:** Primer sequences.

Name of gene	Primer	Sequence (5′–3′)
HIF-1α	Forward	GTC​AGC​AAC​GTG​GAA​GGT​GC
	Reverse	GCA​CCA​AGC​ACG​TCA​TAG​GC
VEGF	Forward	GCA​GTG​CTC​CCC​ATC​CGC​TG
	Reverse	TGC​TCG​TCC​GAC​AGC​TGG​GA
VEGFR-2	Forward	TGG​CAA​TTC​CCG​TCC​TCA​AAG​C
	Reverse	CTT​GGT​CAC​TCT​TGG​TCA​CAC​TGT​C
PI3K	Forward	GCA​ACT​CCT​GGA​CTG​CAA​CT
	Reverse	CAG​CGC​ACT​GTC​ATG​GTA​TG
AKT	Forward	TAG​CCA​TTG​TGA​AGG​AGG​GC
	Reverse	CCT​GAG​GCC​GTT​CCT​TGT​AG
mTOR	Forward	GCT​CCA​GCA​CTA​TGT​CAC​CA
	Reverse	CGT​CTG​AGC​TGG​AAA​CCA​GT
*β*-actin	Forward	CAT​CCT​GCG​TCT​GGA​CCT​GG
	Reverse	TAA​TGT​CAC​GCA​CGA​TTT​CC

#### Western blotting

Total protein was extracted from rat hippocampi using RIPA lysis buffer containing protease- and phosphatase inhibitors. After homogenization, the solution was allowed to stand on ice for 20 min before centrifugation at 13,000 rpm at 4°C for 15 min. After the supernatant was collected, protein concentration was measured using a BCA protein assay kit (catalog number: KGP902, Kaiji Biology, Jiangsu, China). Thereafter, 20 μg protein was separated by 10% sodium dodecyl sulfate-polyacrylamide gel electrophoresis (SDS-PAGE) and then transferred to PVDF membranes (0.45 μm, Millipore, United States) at 300 mA for 1.5 h. The membranes were blocked with 5% skim milk for 1.5 h and then incubated with the primary antibodies at 4°C overnight. After washing with TBST thrice, the membranes were then incubated with secondary antibodies at room temperature for 1 h. Subsequently, the membranes were developed using the Ultrasensitive ECL Chemiluminescence Kit (catalog number: P10100, NCM, Jiangsu, China). The labeled proteins were detected using a fully automated chemiluminescence image analysis system (Tanon-5200, Shanghai, China). Gray value analysis was performed using the ImageJ software (The National Institutes of Health, USA).

### Molecular docking

Three-dimensional structures of target proteins associated with depression, including HIF-1α, VEGF, and VEGFR-2, were downloaded from the RCSB PDB database (https://www.rcsb.org/). The crystal structures were selected from the species “Homo sapiens”, with a resolution of < 3 and intact binding sites. This was then saved as a PDB format. Water molecules and small molecule ligands were removed from the protein structures using the PyMol 2.4.1 software (Delano Scientific LLC, Italy). The AutoDockTools software was used to add hydrogen and finally convert to the PDBQT format. The two-dimensional structure of honokiol was obtained from the PubChem database (https://pubchem.ncbi.nlm.nih.gov/) and saved in the SDF format. Afterwards, the SDF format was converted to the Mol2 format using the Open Bable 2.4.1 software. Finally, the AutoDockTools software was used to assign charges, detect rotatable keys, and save them as PDBQT format files. The AutoDock 4.2.6 software (The Scripps Research Institute, USA) was used to perform molecular docking analysis on targets and active ingredients. Visualization was performed using the PyMol 2.4.1 software.

### Statistical analysis

The experimental data are expressed as the mean ± standard error of the mean (SEM). GraphPad Prism 9 (GraphPad Software Inc, San Diego, CA, United States) and SPSS 20 (SPSS, Chicago, Illinois, United States) were used for statistical analysis and graphing. Data with normal distribution and homogeneity of variance were analyzed using one-way ANOVA followed by an LSD post hoc test. *p* > 0.05 was considered normally distributed. The Kruskal-Wallis test was used for data with non-normal distribution. Differences were statistically significant when *p* < 0.05.

## Results

### Effects of honokiol on PC12 cells

#### Effects of honokiol on the viability of normal PC12 cells

**FIGURE 3:**
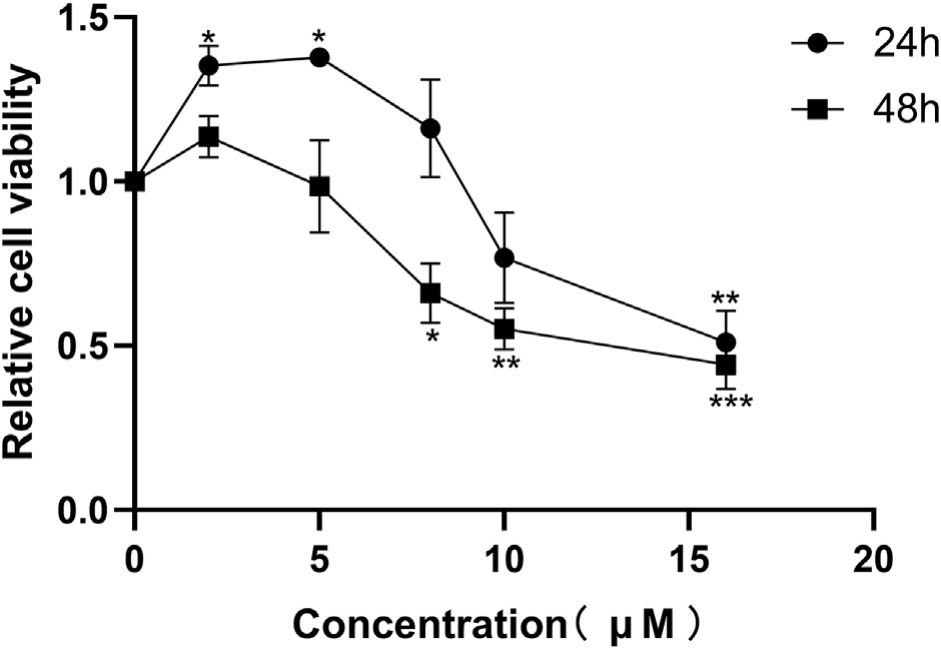
Effects of honokiol on the viability of normal PC12 cells. Data are expressed as mean ± SEM (*n* = 3). ^*^ *p* < 0.05 vs. Con group. ^**^ *p* < 0.01 vs. Con group. ^***^ *p* < 0.001 vs. Con group.

First, this study screened the concentration and time of honokiol administration. PC12 cells were exposed to different concentrations of honokiol for 24 and 48 h. After honokiol treatment for 24 h, PC12 cell viability was observed to have a significant increase at 2 and 5 μM and a marked decrease at 16 μM, compared to the control group (Figure 3; F_5,12_ = 12.678; ^*^ *p* < 0.05; ^**^ *p* < 0.01). Additionally, the cell viability of 5 μM honokiol was not significantly different from than of the control group at 48 h. Cell viability began to decrease significantly when the concentration of honokiol was > 8 μM (Figure 3; F_5,12_ = 11.497; ^*^ *p* < 0.05; ^**^ *p* < 0.01; ^***^ *p* < 0.001). Furthermore, the cell viability at 16 μM honokiol for 24 and 48 h were 51.0 ± 16.9% (F_5,12_ = 12.678, ^**^ *p* < 0.01) and 44.3 ± 12.9% (F_5,12_ = 11.497, ^***^ *p* < 0.001). Additionally, the cell viability was as high as 135.4 ± 10.6% for 24 h at 2 μM honokiol, which showed a significant pro-proliferative effect, and showed no significant difference from that of 5 and 8 μM honokiol. Therefore, 2 μM honokiol for 24 h incubation was chosen as the concentration and time of honokiol administration.

#### Effects of SU5416 and 2-ME on synaptic plasticity of neurons in PC12 cells

**FIGURE 4:**
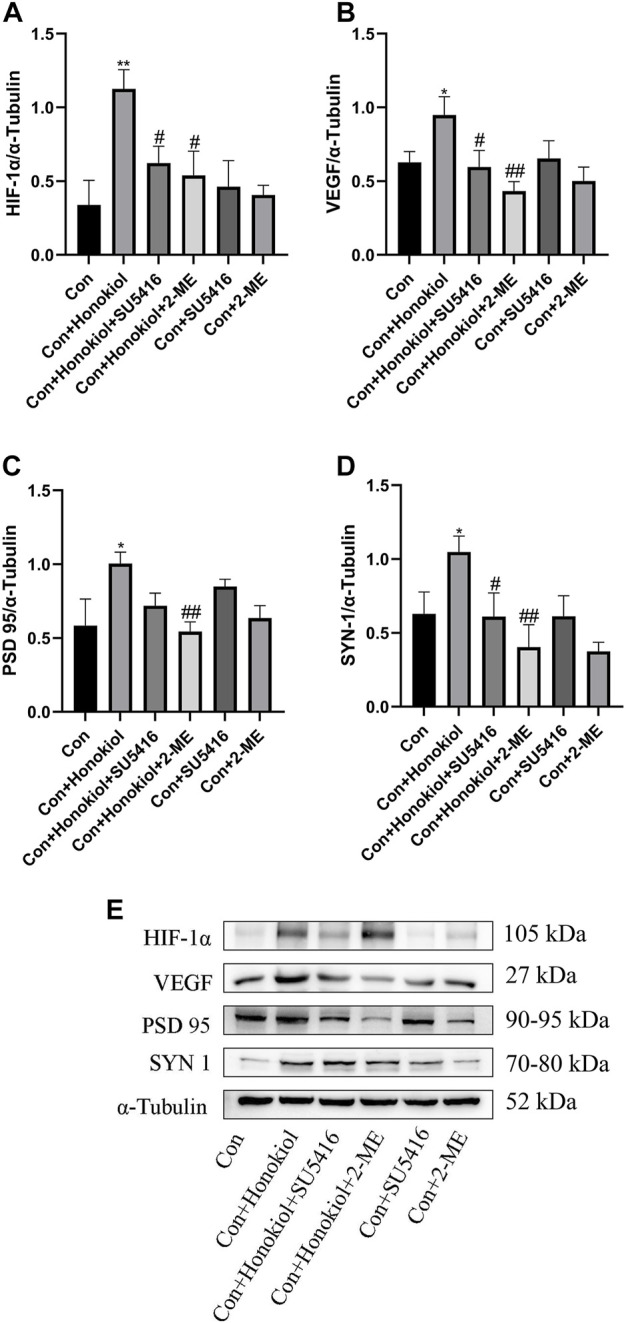
Effects of SU5416 and 2-ME on synaptic plasticity of neurons in PC12 cells. **(A)** HIF-1α protein expression. **(B)** VEGF protein expression. **(C)** PSD 95 protein expression. **(D)** SYN 1 protein expression. **(E)** The Western blot bands. Data are expressed as mean ± SEM (*n* = 3). ^*^ *p* < 0.05 vs. Con group. ^**^ *p* < 0.01 vs. Con group. ^#^ *p* < 0.05 vs. Con + Honokiol group. ^##^ *p* < 0.01 vs. Con + Honokiol group.

To investigate the mechanism of the antidepressant-like effect of honokiol, we used 2-ME and SU5416 to explore whether the neuronal synaptic plasticity effect of honokiol was dependent on the HIF-1α-VEGF signaling pathway. HIF-1α (F_5,12_ = 4.05, *p* = 0.002), VEGF (F_5,12_ = 3.121, *p* = 0.044), PSD 95 (F_5,12_ = 3.121, *p* = 0.011), and SYN-1(F_5,12_ = 3.317, *p* = 0.045) protein expressions were significantly higher after honokiol treatment, compared to the control group (Figure 4). Compared with the Con + Honokiol group, HIF-1α (F_5,12_ = 4.05, *p* = 0.012), VEGF (F_5,12_ = 3.121, *p* = 0.004), PSD 95 (F_5,12_ = 3.121, *p* = 0.007), and SYN 1 (F_5,12_ = 3.317, *p* = 0.005) protein expressions were significantly lower after 2-ME treatment (Figure 4). Additionally, HIF-1α (F_5,12_ = 4.05, *p* = 0.027), VEGF (F_5,12_ = 3.121, *p* = 0.03), and SYN 1 (F_5,12_ = 3.317, *p* = 0.038) protein expressions in the Con + Honokiol + SU5416 group were significantly decreased (Figure 4), without any significant difference in PSD 95 protein expression. Based on these results, honokiol was shown to enhance synaptic plasticity in PC12 cells by activating the HIF-1α-VEGF signaling pathway. The blockers played an inhibitory role. Moreover, HIF-1α, VEGF, PSD 95, and SYN 1 protein expressions in cells treated with 2-ME and SU5416 only did not differ from those of the control group, which excluded the possibility that 2-ME and SU5416 mediated interference.

### Effects of honokiol in chronic unpredictable mild stress depression rat models

#### Effects of honokiol in open field test

**FIGURE 5:**
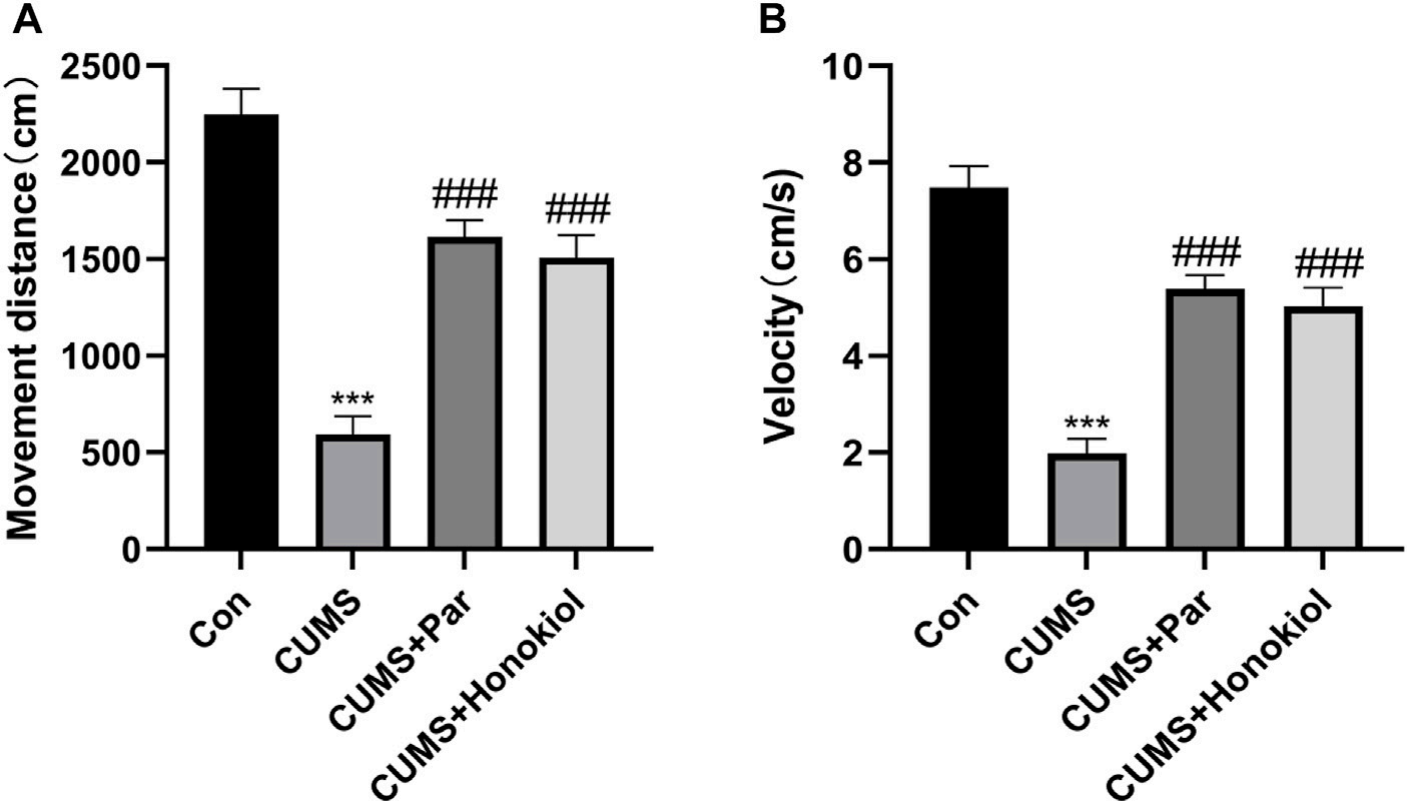
Effects of honokiol in OFT. **(A)** The movement distance. **(B)** The movement velocity. Data are expressed as mean ± SEM (*n* = 8/group). ^***^ *p* < 0.001 vs. Con group. ^###^ *p* < 0.001 vs. CUMS group.

The OFT was used to evaluate the spontaneous activity of rats. The distance and velocity of movement of the CUMS group were lower than those of the control group (Figure 5. F_3,28_ = 39.532, ^***^ *p* < 0.001). However, the distance and velocity of movement of the honokiol treatment group were markedly higher than those of the CUMS group (Figure 5. F_3,28_ = 39.532, ^###^ *p* < 0.001).

#### Effects of honokiol in sucrose preference test

**FIGURE 6:**
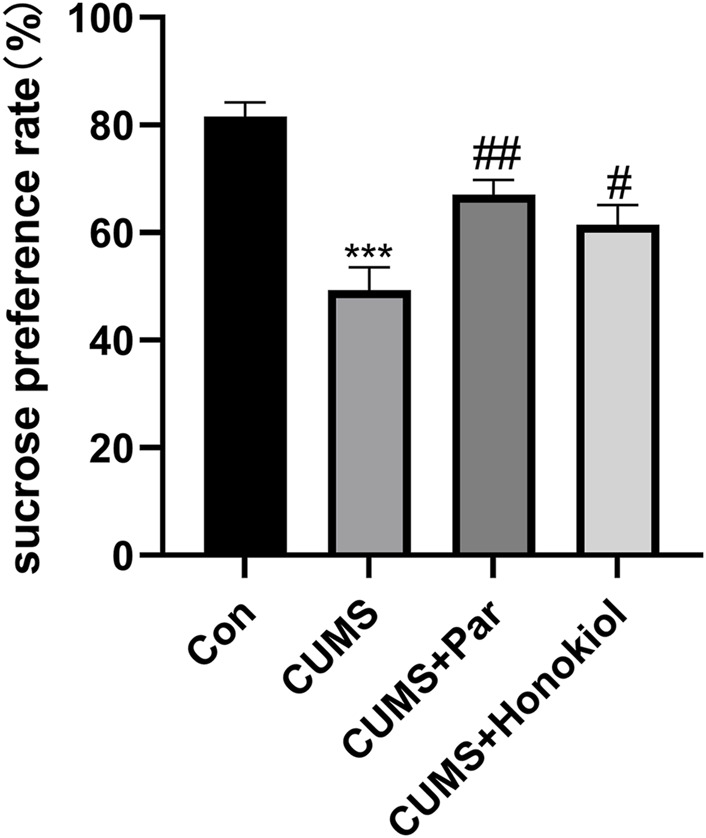
Effects of honokiol in SPT. Data are expressed as mean ± SEM (*n* = 8/group). ^***^ *p* < 0.001 vs. Con group. ^#^ *p* < 0.05 vs. CUMS group.

The sucrose preference test was used to assess motivation, emotional state, and pleasure-deficit behavior in rats. Compared with the control group, the CUMS group had a decreased sucrose preference rate (Figure 6. F_3,28_ = 15.638, ^***^ *p* < 0.001). By contrast, the sucrose preference rate was elevated after treatment with honokiol (Figure 6. F_3,28_ = 15.638, ^#^ *p* < 0.05). No significant difference in the rate of sucrose preference was observed between the honokiol and paroxetine groups. The results suggest that honokiol reversed CUMS-induced anhedonia.

#### Effects of honokiol on the HIF-1α-VEGF signaling pathway in the hippocampus

**FIGURE 7:**
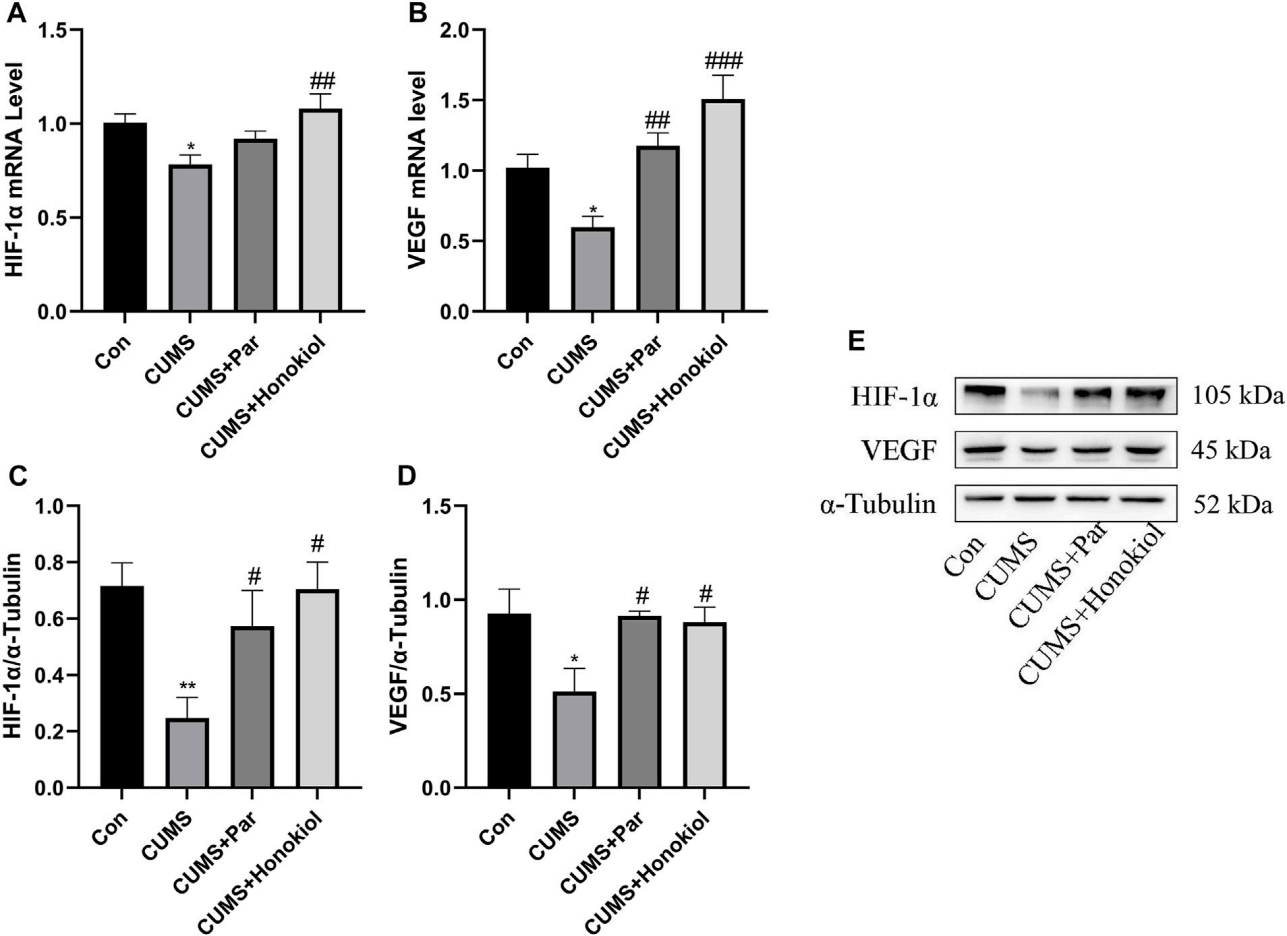
Effects of honokiol on the HIF-1α-VEGF signaling pathway in the hippocampus. **(A)** HIF-1α mRNA expression. **(B)** VEGF mRNA expression. Data are expressed as mean ± SEM (*n* = 6/group). **(C)** HIF-1α protein expression. **(D)** VEGF protein expression. **(E)** The Western blot bands. Data are expressed as mean ± SEM (*n* = 3/group). ^*^ *p* < 0.05 vs. Con group. ^**^ *p* < 0.01 vs. Con group. ^#^ *p* < 0.05 vs. CUMS group. ^##^ *p* < 0.01 vs. CUMS group. ^###^ *p* < 0.001 vs. CUMS group.

The mRNA and protein expression levels of HIF-1α and VEGF were detected by real-time PCR and western blot technologies. HIF-1α and VEGF mRNA and protein expression were obviously lower in the CUMS group than in the control group (Figure 7. HIF-1α protein: F_3,8_ = 5.127, *p* = 0.009; VEGF protein: F_3,8_ = 4.055, *p* = 0.018; HIF-1α mRNA: F_3,20_ = 5.286, *p* = 0.01; VEGF mRNA: F_3,20_ = 10.945, *p* = 0.016). Contrarily, honokiol significantly increased the expression levels of HIF-1α and VEGF mRNA and protein in the CUMS rat models (Figure 7. HIF-1α protein: F_3,8_ = 5.127, *p* = 0.01; VEGF protein: F_3,8_ = 4.055, *p* = 0.03; HIF-1α mRNA: F_3,20_ = 5.286, *p* = 0.001; VEGF mRNA: F_3,20_ = 10.945, *p* < 0.001).

#### Effects of honokiol on mRNA and protein expression of VEGFR-2 in the hippocampus

**FIGURE 8:**
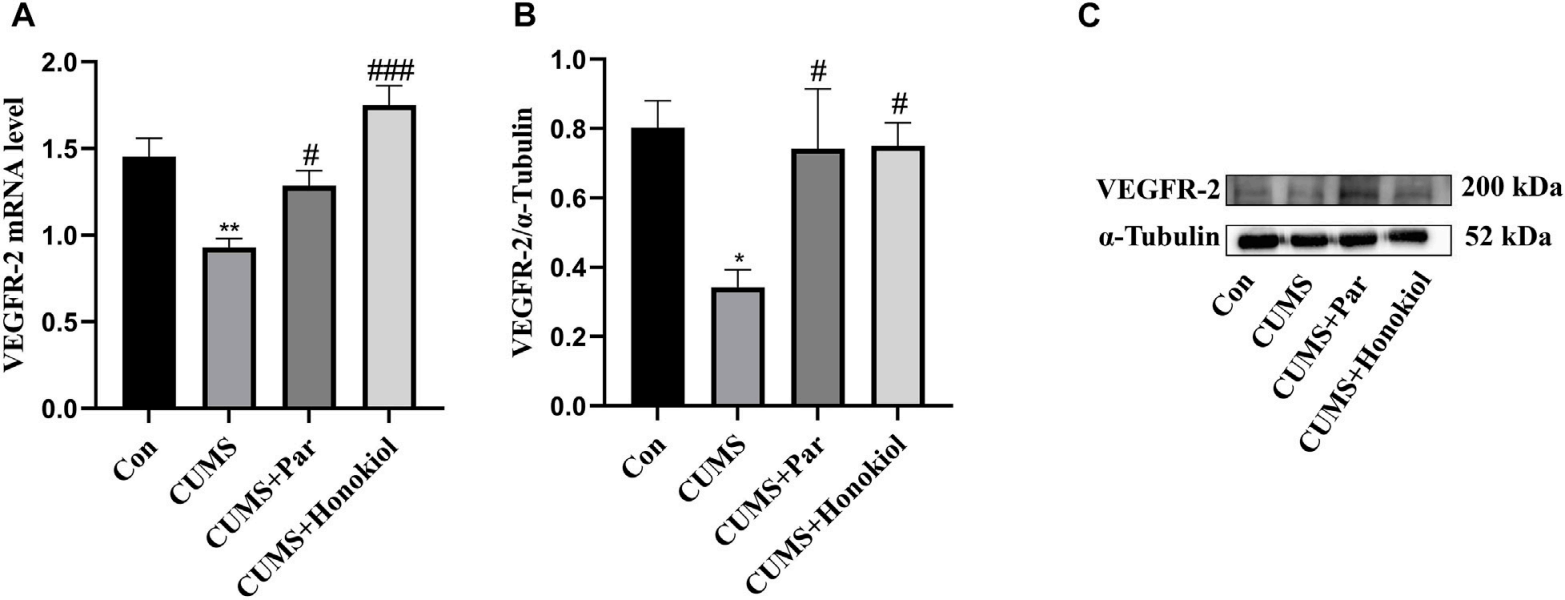
Effects of honokiol on mRNA and protein expression of VEGFR-2 in the hippocampus. **(A)** VEGFR-2 mRNA expression. Data are expressed as mean ± SEM (*n* = 6/group). **(B)** VEGFR-2 protein expression. **(C)** The Western blot bands. Data are expressed as mean ± SEM (*n* = 3/group). ^*^ *p* < 0.05 vs. Con group. ^**^ *p* < 0.01 vs. Con group. ^#^ *p* < 0.05 vs. CUMS group. ^###^ *p* < 0.001 vs. CUMS group.

By detecting the expression of VEGFR-2 mRNA and protein using Real-time PCR and western blot, it was explored whether honokiol exerted an antidepressant-like effect by acting on VEGFR-2. Data demonstrated that VEGFR-2 mRNA and the protein expression level were remarkably reduced in the CUMS group compared to the control group (Figure 8. VEGFR-2 protein: F_3,8_ = 4.234, *p* = 0.014; VEGFR-2 mRNA: F_3,20_ = 13.855, *p* = 0.001). However, VEGFR-2 mRNA and protein expression were significantly higher after honokiol treatment (Figure 8. VEGFR-2 protein: F_3,8_ = 4.234, *p* = 0.024; VEGFR-2 mRNA: F_3,20_ = 13.855, *p* < 0.001).

#### Effects of honokiol on mRNA and protein expression of PI3K/AKT/mTOR pathway in the hippocampus

**FIGURE 9:**
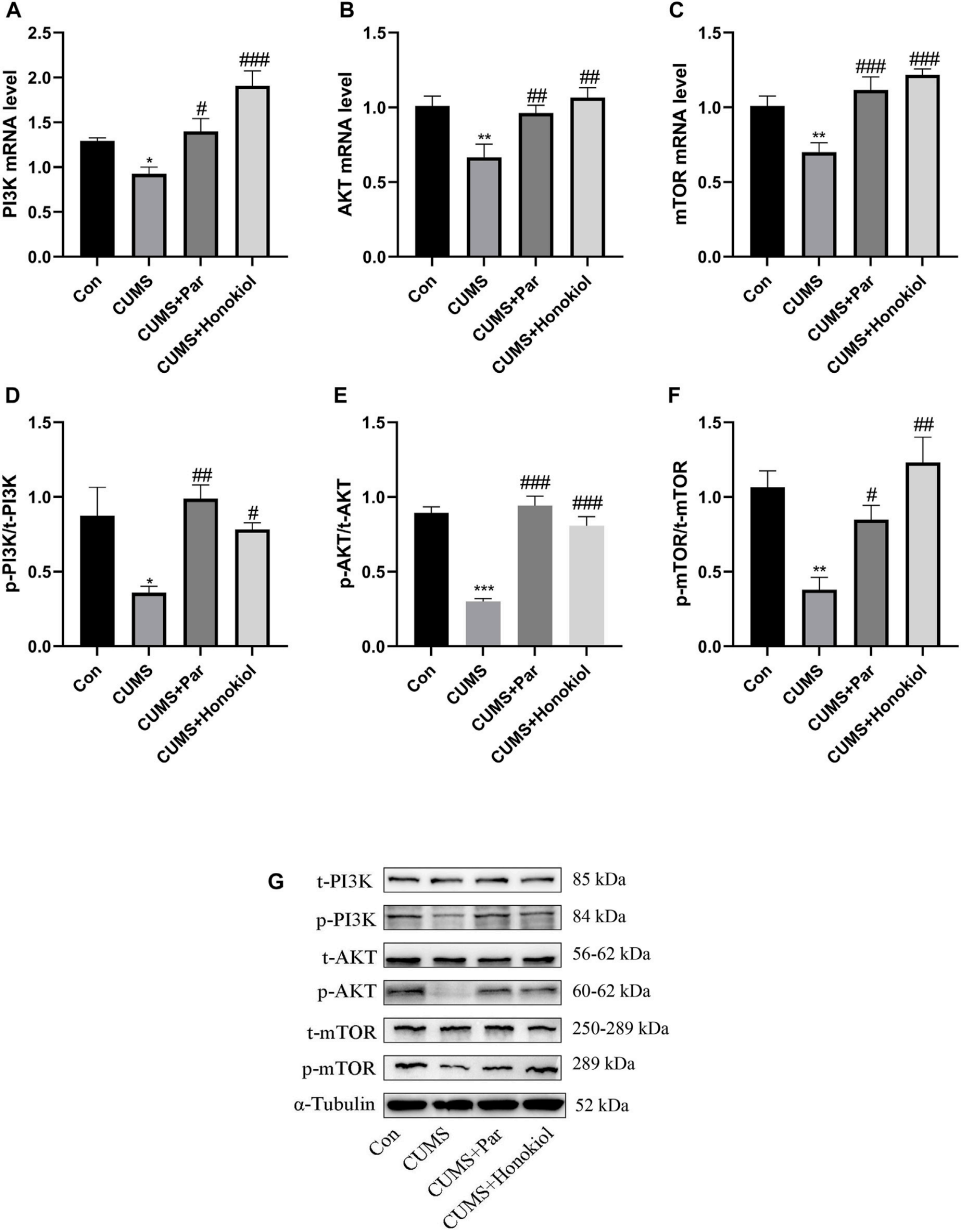
The effects of honokiol on PI3K/AKT/mTOR signaling pathway in the hippocampus. **(A)** PI3K mRNA expression. **(B)** AKT mRNA expression. **(C)** mTOR mRNA expression. Data are expressed as mean ± SEM (n = 6/group). **(D)** p-PI3K protein expression. **(E)** p-AKT protein expression. **(F)** p-mTOR protein expression. **(G)**The Western blot bands. Data are expressed as mean ± SEM (*n* = 3/group). ^*^ *p* < 0.05 vs. Con group. ^**^ *p* < 0.01 vs. Con group. ^***^ *p* < 0.001 vs. Con group. ^#^ *p* < 0.05 vs. CUMS group. ^##^ *p* < 0.01 vs. CUMS group. ^###^ *p* < 0.001 vs. CUMS group.

This experiment was conducted to explore whether the PI3K/Akt/mTOR signaling pathway was involved in the antidepressant-like effects of honokiol. PI3K, AKT, mTOR mRNA, and p-PI3K, p-AKT, and p-mTOR protein expressions were significantly downregulated in the hippocampus of CUMS rat models, compared with the control group (Figure 9. p-PI3K protein: F_3,8_ = 6.226, *p* = 0.011; p-AKT protein: F_3,8_ = 35.534, *p* < 0.001; p-mTOR protein: F_3,8_ = 9.638, *p* = 0.004; PI3K mRNA: F_3,20_ = 12.077, *p* = 0.038; AKT mRNA: F_3,20_ = 6.717, *p* = 0.002; mTOR mRNA: F_3,20_ = 11.456, *p* = 0.004). Additionally, honokiol significantly elevated PI3K, AKT, and mTOR mRNA and p-PI3K, p-AKT, and p-mTOR protein expression in CUMS rat models (Figure 9. p-PI3K protein: F_3,8_ = 6.226, *p* = 0.026; p-AKT protein: F_3,8_ = 35.534, *p* < 0.001; p-mTOR protein: F_3,8_ = 9.638, *p* = 0.001; PI3K mRNA: F_3,20_ = 12.077, *p* < 0.001; AKT mRNA: F_3,20_ = 6.717, *p* = 0.001; mTOR mRNA: F_3,20_ = 11.456, *p* < 0.001). Therefore, the results confirmed that honokiol improved CUMS-induced depressive behaviors in rats *via* the PI3K/AKT/mTOR pathway.

#### Effects of honokiol on regulators related to synaptic plasticity in the hippocampus

**FIGURE 10:**
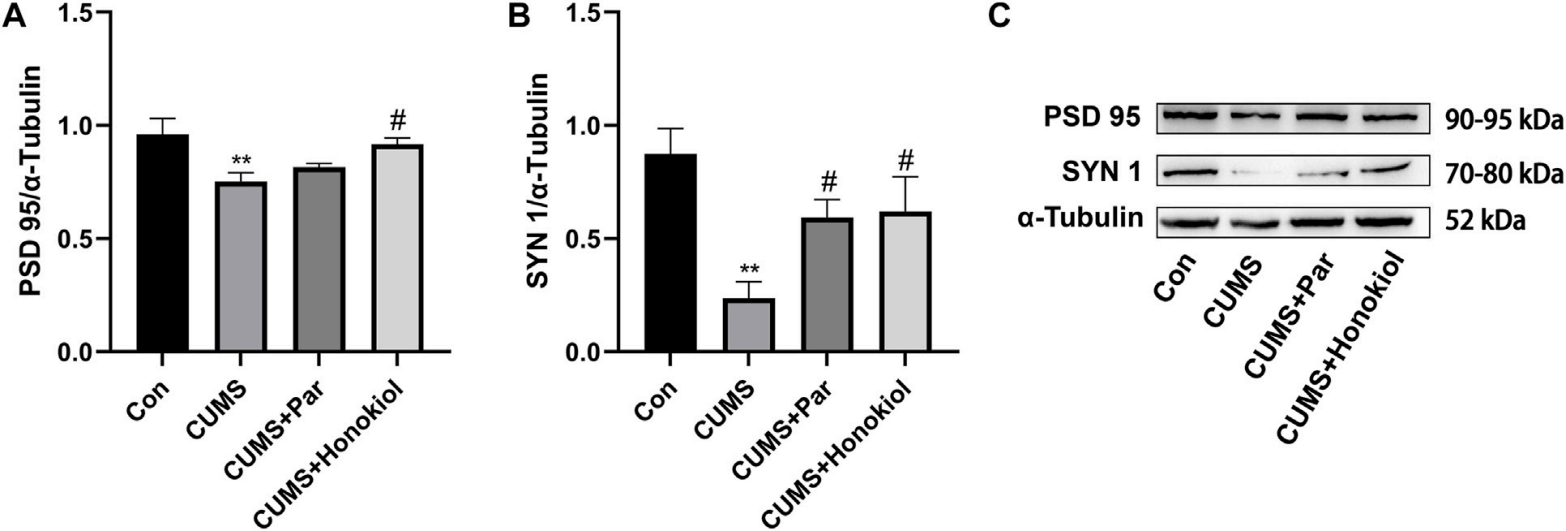
Effects of honokiol on regulators related to synaptic plasticity in the hippocampus. **(A)** PSD 95 protein expression. **(B)** SYN 1 protein expression. **(C)** The Western blot bands. Data are expressed as mean ± SEM (*n* = 3/group). ^**^ *p* < 0.01 vs. Con group. ^#^ *p* < 0.05 vs. CUMS group.

In addition to studying the synaptic plasticity action of honokiol on PC12 cells, the experiment explored whether honokiol exerted an antidepressant effect in CUMS rat models by modulating synaptic plasticity-related regulators. Data indicated that PSD 95 (F_3,8_ = 4.936, *p* = 0.009) and SYN 1 (F_3,8_ = 5.893, *p* = 0.003) protein expressions were significantly lower in the CUMS group than those of the control group (Figure 10). PSD 95 (F_3,8_ = 4.936, *p* = 0.026) and SYN 1(F_3,8_ = 5.893, *p* = 0.036) protein expression levels were elevated after honokiol treatment (Figure 10).

### Molecular docking results

**FIGURE 11:**
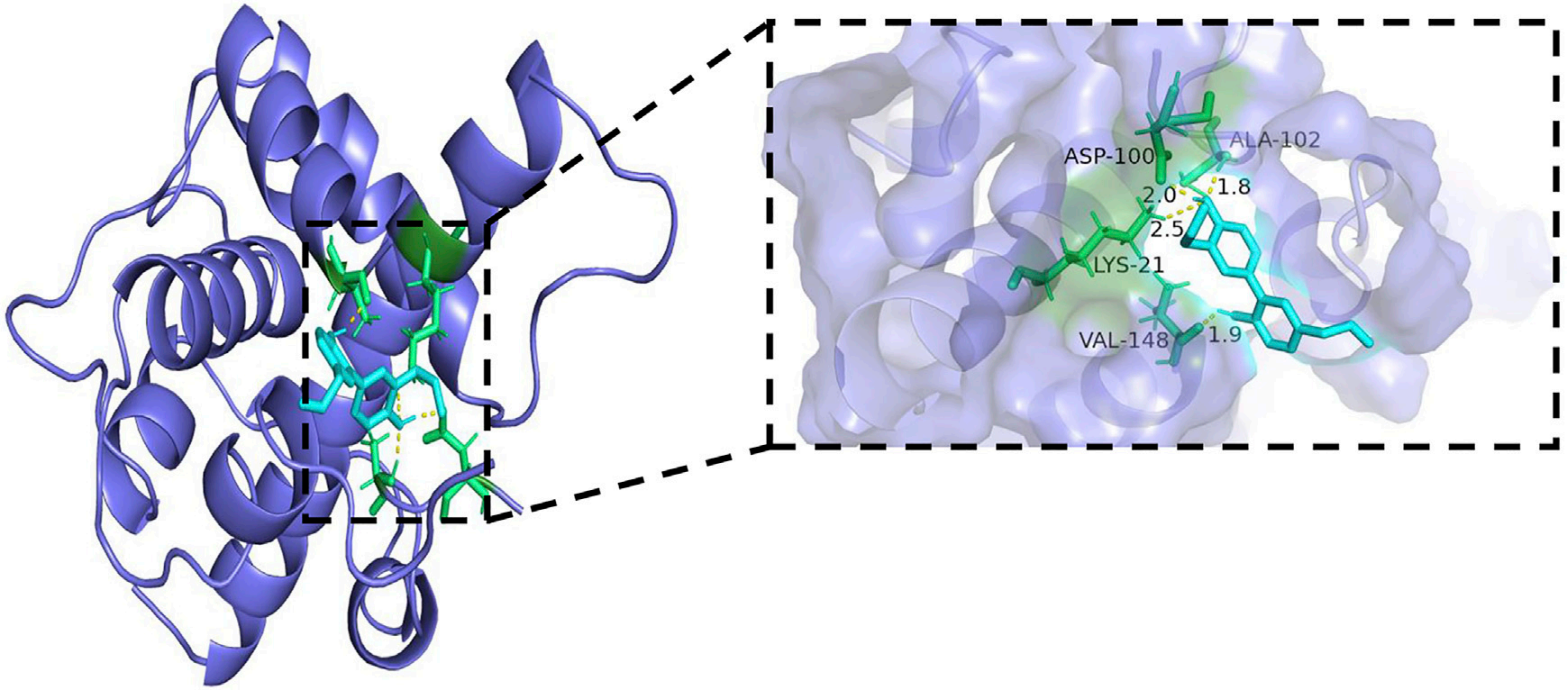
Molecular docking schema of Honokiol and HIF-1α.

**FIGURE 12:**
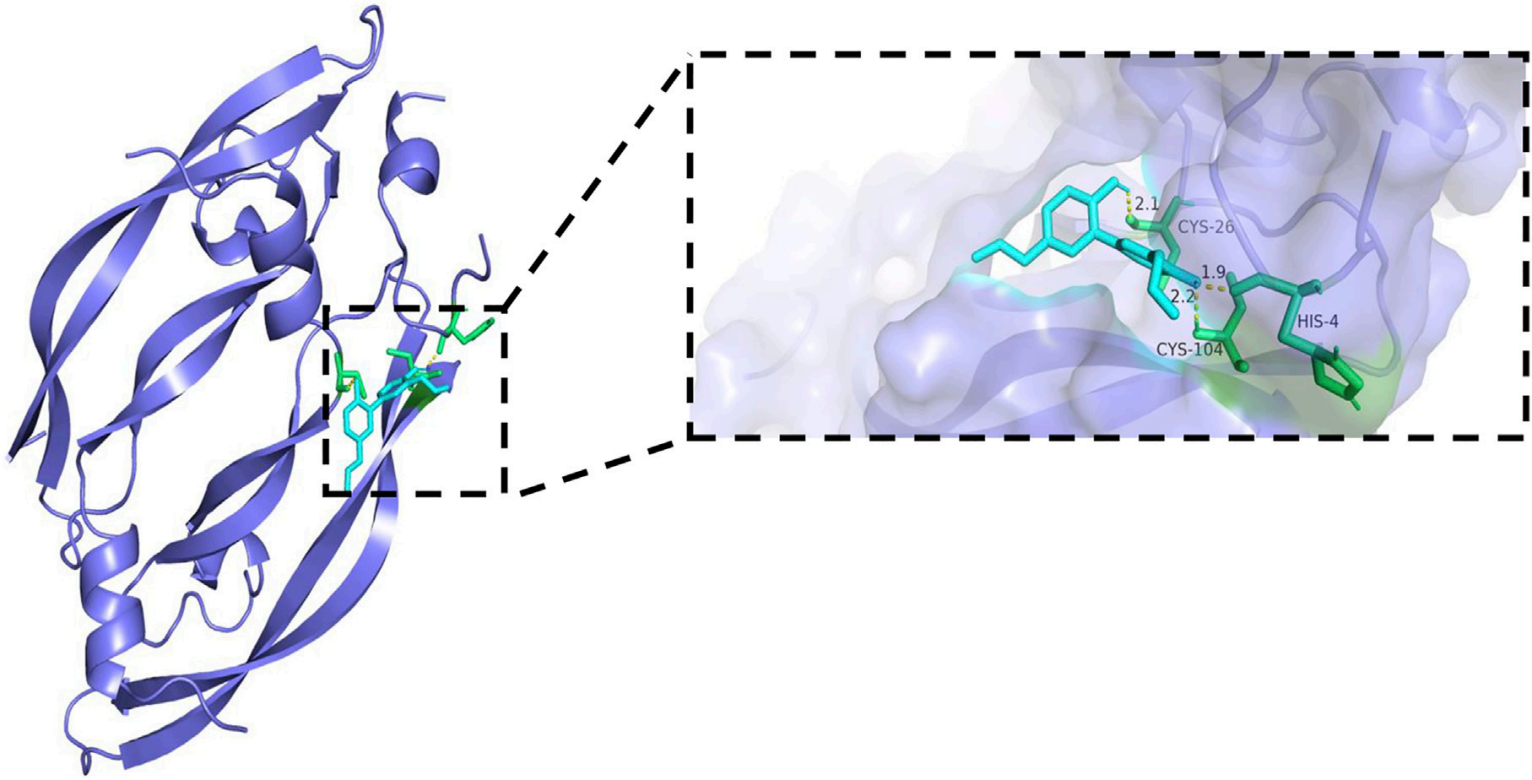
Molecular docking schema of Honokiol and VEGF.

**FIGURE 13:**
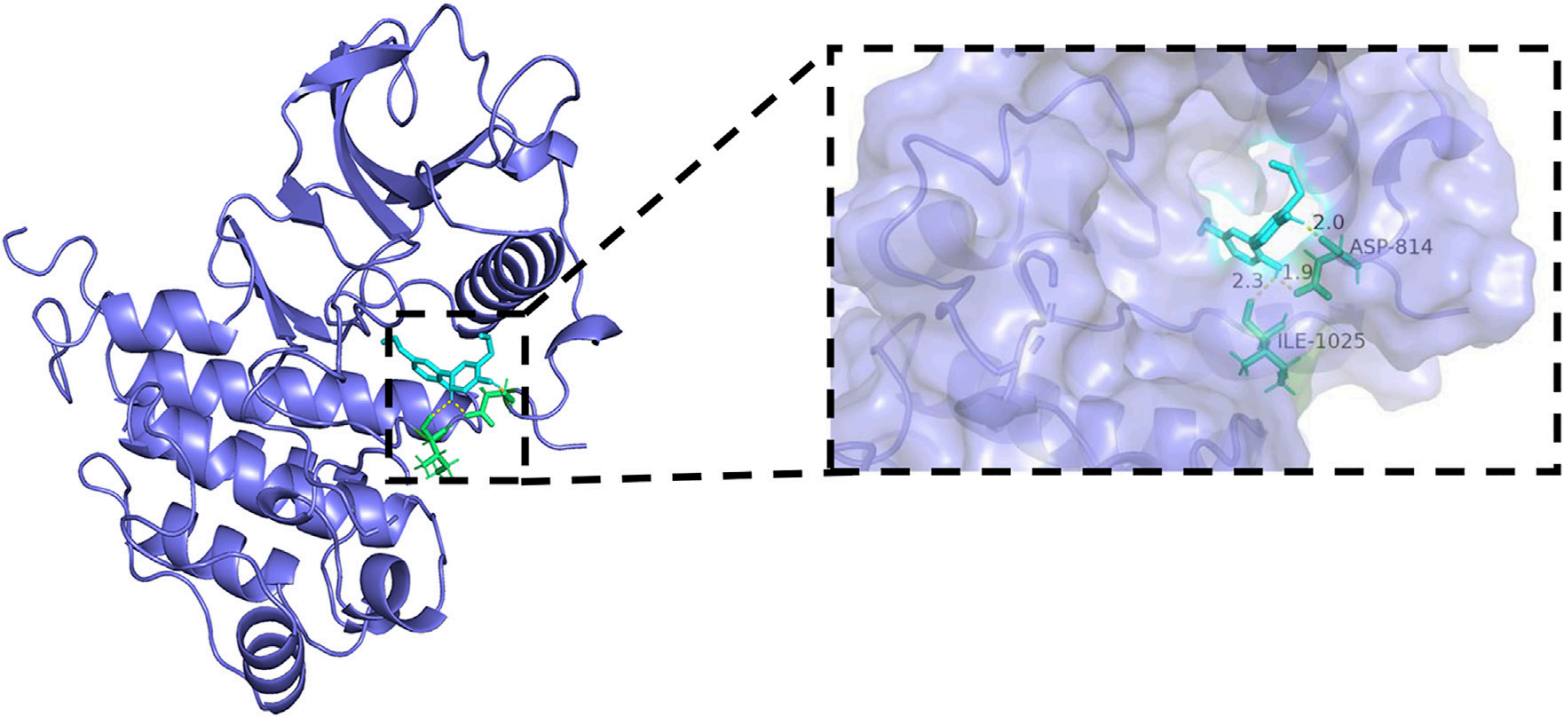
Molecular docking schema of Honokiol and VEGFR-2.

Generally, lower binding energy between ligand and receptor is considered to result in more stable binding conformation and greater possibility of interaction. The binding energies between core target protein receptors (HIF-1α, VEGF, and VEGFR-2) and a small molecule ligand (honokiol) were −6.01, −6.49, and −6.1 kcal/mol, respectively. The molecular docking binding energies were all < −5 kcal/mol, which indicated high binding activity and stable docking. Hydrogen bonding is the main force that drives ligand binding to the active site. Honokiol can form hydrogen bonds with active sites, ASP-100, LYS-21, VAL-148, and ALA-102, of the HIF-1α gene encoding protein (PDB:1L8C). Furthermore, it can form hydrogen bonds with active sites, CYS-26, CYS-104, and HIS-4, of the VEGF gene encoding protein (PDB:6z3f). It can form hydrogen bonds with active sites, ASP-814 and, ILE-1025, of the VEGFR-2 gene encoding protein (PDB:3VHE). The molecular docking schematics of honokiol and core targets are shown in Figures 11–13.

## Discussion

In this study, we aimed to demonstrate whether honokiol has antidepressant-like properties by activation of the HIF-1α-VEGF signaling pathway. The study of antidepressant mechanisms using 2-ME and SU5416 *in vitro* showed that honokiol enhanced synaptic plasticity in PC12 cells, which relied on the activation of the HIF-1α-VEGF signaling pathway. *In vivo*, honokiol could reverse CUMS-induced depression-like behaviors. Honokiol activated the HIF-1α-VEGF signaling pathway, regulated the PI3K-AKT-mTOR signaling pathway mediated by VEGFR-2, and increased the expression of synaptic plasticity regulators. We believe these findings collectively demonstrate that honokiol is a promising and effective drug for the treatment of depression, and HIF-1α-VEGF pathway is a promising target.

The PC12 cells used in this study, a common neural cell strain, were derived from rat adrenal pheochromocytoma. PC12 cells were differentiated into neurons. As shown in Figure 2, morphologically differentiated PC12 cells have multiple neurites and long synapses. Additionally, MAP2 expression can be observed by immunofluorescence to identify whether PC12 cells differentiated into neurons (Yi et al., 2021). PC12 cells are close to nerve cells in terms of morphology, physiology, and biochemical function, and have the characteristics of passage (Tischler and Greene, 1975). PC12 cells have become a tool cell for studying neurophysiology and neuropharmacology *in vitro*. Using various techniques to study differentiated PC12 cells is a direction needed for future studies.

The CUMS depression model is a reliable and effective rodent model of depression (Antoniuk et al., 2019). Various environmental stimuli are applied to animals for a long time under unpredictable conditions, inducing animals to produce various long-term physiological, behavioral, neuroendocrine, and other changes, which can effectively simulate all kinds of psychological pressures in patients with depression (Leonard and Maes, 2012). The results of the SPT and OFT showed that the CUMS model was successfully established. Honokiol improved autonomic activity and reversed the loss of pleasure in CUMS rats, indicating an effective antidepressant action.

HIF-1, a cellular oxygen sensor, is upregulated during tissue hypoxia to protect cells from hypoxia-induced dysfunction, whereas it is rapidly degraded under normoxic conditions (Ke and Costa, 2006; Kaelin and Ratcliffe, 2008). Many target genes are regulated by HIF-1 and mediate protein synthesis through HIF-1 binding to hypoxia response elements (HREs) of the target genes (Semenza, 2012). Intermittent hypoxia (IH) has been reported to stimulate hippocampal angiogenesis and neurogenesis, enhance nerve cell proliferation, and improve brain memory impairment (Bouslama et al., 2015). Additionally, IH has antidepressant-like effects in various animal models of depression, such as behavioral despair models and chronic mild stress models (Zhu et al., 2010). Specifically, this effect is achieved by improving the expression of HIF-1 and its target genes, EPO and VEGF. Most importantly, EPO and VEGF are sufficient to produce robust antidepressant effects in animal models (Deyama et al., 2019).

VEGF is a physiologically powerful mitogen of endothelial cells (Kim et al., 2008). In addition to its pro-angiogenic activity, recent studies have revealed that VEGF has neurotrophic and neuroprotective potential in the central nervous system (CNS) (Rosenstein et al., 2003). VEGF affects neuronal plasticity in the CNS and promotes axonal growth and neurogenesis (Tillo et al., 2012). Long-term cognitive deficits and a decrease in VEGF level have been observed in patients with depression (Viikki et al., 2010). In the treatment of depression, some antidepressants increase VEGF levels and promote nerve cell proliferation (Warner-Schmidt and Duman, 2007). As a neurotrophic factor, VEGF has become a hot spot in the study of psychiatric disorders and the effects of psychotropic drugs (Howell and Armstrong, 2017). VEGFR-2, a cell surface receptor for VEGF, can exert antidepressant-like effects by interacting with VEGF (De Rossi et al., 2016). VEGF plays a significant role in learning and memory, which specifically reflects on long-term enhancement, plasticity improvement, and cognitive function improvement mediated by its receptor VEGFR-2 (Yang et al., 2014). Additionally, chronic stress in the dentate gyrus of the adult hippocampus reduces cell proliferation near blood vessels and the expression of VEGF and VEGFR-2 proteins (Heine et al., 2005). Based on the complexity of the pathogenesis of depression, VEGF and VEGFR-2 signals deserve further investigation as potential targets for antidepressant therapy (Kenwood et al., 2019). In summary, the HIF-1α-VEGF signaling pathway is closely related to the treatment of depression, which is consistent with our experimental results. Honokiol significantly increased mRNA and protein expression of HIF-1α, VEGF, and VEGFR-2 in the CUMS model group.

2-ME, an anti-angiogenic, antiproliferative, and pro-apoptotic agent, is a natural metabolite of estradiol and an effective HIF-1α inhibitor (Aquino-Gálvez et al., 2016). 2-ME inhibits the transcriptional activity and protein expression of HIF-1α by inducing microtubule depolymerization and the production of reactive oxygen species (Khan et al., 2015; Khan et al., 2018). SU5416 is a potent and selective inhibitor of VEGFR-2. Angiogenesis inhibitor, SU5416, inhibits VEGFR-2 expression by inhibiting endothelial cell mitosis, tyrosine kinase catalysis, and microcirculation (Vajkoczy et al., 1999; Zagorski et al., 2022). Besides, *in vivo* and *in vitro* experiments have shown that VEGF activates Erk1/2 and AKT signaling pathways in adult rat hippocampi and hippocampal neuronal precursor cells, and this effect is blocked by SU5416 (Fournier et al., 2012). SU5416 has been widely used in hippocampal nerve-related diseases. Our experimental findings demonstrated that the neuroplasticity effect of honokiol was blocked by 2-ME and SU5416, supporting the conclusion that the beneficial effect of honokiol was dependent on the HIF-1α-VEGF pathway.

Currently, numerous studies have been conducted on the downstream pathways of VEGFR-2. Studies have shown that the PI3K-AKT-mTOR signaling pathway mediated by VEGFR-2 can regulate neuroplasticity (Cao et al., 2017). PI3K receives extracellular stimulation to initiate downstream AKT/mTOR, which promotes neurogenesis, neural cell proliferation, and synaptic plasticity (Fakhri et al., 2021). The activity of this pathway was significantly reduced in depressed states. Consistent with our experimental results, the expression levels of PI3K-AKT-mTOR pathway-related genes and proteins decreased in CUMS depression rat models.

Mammalian target of rapamycin (mTOR), a serine-threonine kinase, regulates various cellular functions in mammals. mTOR signaling is closely related to synaptic plasticity and can improve impairment of synaptic plasticity in multiple ways (Xia et al., 2021). Nuclear factor-kappa B (NF-κB), an important transcription factor, regulates the expression of many immune and inflammatory factors (Hoffmann and Baltimore, 2006). The NF-κB signaling pathway plays an important role in depression-like behaviors induced by acute and chronic stress and lipopolysaccharides (Munhoz et al., 2006; Koo et al., 2010). The mTOR pathway and its downstream factor, NF-κB, have been found to play a crucial role in maintaining normal physiological functions of the nervous system, especially for learning and cognitive functions (Dan et al., 2008; Lu et al., 2011). Additionally, mTOR is activated to regulate its target gene, NF-κB, under hypoxia, thus affecting cell growth, metabolism, proliferation, and differentiation (Jiang et al., 2010; Dhingra et al., 2013). Studies have shown that the pathogenesis of depression is closely related to the inflammatory response (Raison et al., 2006). Honokiol can improve inflammation-induced depression-like behaviors. Our previous study found that honokiol improved depression-like behaviors in LPS-induced depression mouse models by inhibiting the NF-κB signaling pathway and reducing the levels of pro-inflammatory cytokines (Zhang et al., 2019). Besides, honokiol can exert anti-inflammatory effects through various pathways, such as inhibition of the PI3K/Akt pathway (Kim and Cho, 2008) and; inhibition of the activation of the NF-κB signaling pathway through the inhibition of IκB kinase (IKK) activities (Tse et al., 2005).

**FIGURE 14:**
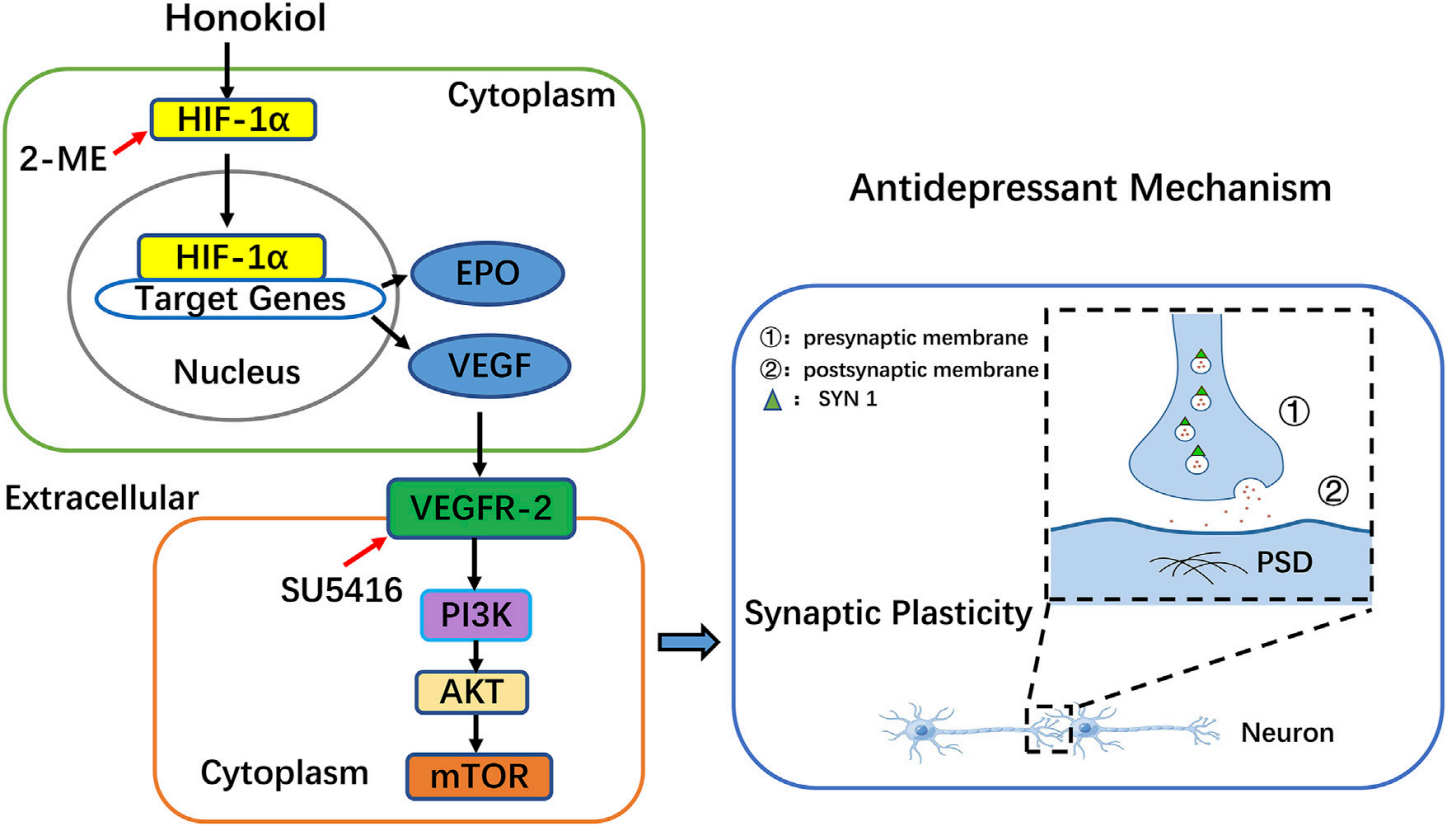
The antidepressant mechanism of Honokiol. Honokiol activates HIF-1α and translocates HIF-1α into the nucleus. Target genes, such as vascular endothelial growth factor (VEGF) and erythropoietin (EPO), are regulated by hypoxia-inducible factor-1α (HIF-1α). HIF-1α can bind to the hypoxia response elements of many target genes to mediate protein synthesis. Ultimately, these growth factors are secreted into the extracellular space between cells. VEGF binds to and activates its receptor VEGFR-2 on adjacent cells. Activation of the VEGFR-2 receptor leads to activation of the PI3K-AKT-mTOR signaling pathway in adjacent cells. The PI3K-AKT-mTOR signaling pathway plays an important role in nerve cell proliferation, survival, and synaptic plasticity, and finally improves depression. 2-ME and SU5416 were applied to investigate whether synaptic plasticity in neurons is dependent on the HIF-1α-VEGF pathway.

Synaptic plasticity is a characteristic of synapses that undergo more lasting changes in morphology and function. Additionally, it is the basis for the recovery of learning, memory, and sensory dysfunction (Ruggiero et al., 2021). The pathogenesis of depression was previously reported to be associated with synaptic plasticity disorders (Pilar-Cuéllar et al., 2013; Li et al., 2020b). Our previous studies have shown that honokiol exhibits significant antidepressant-like effects by affecting tryptophan metabolism and reducing the serum levels of corticosterone to improve neuronal plasticity (Zhang et al., 2019; Zhang et al., 2020). Furthermore, honokiol was shown to bind to neural cell adhesion molecules to enhance neuronal survival and synaptic plasticity (Xu et al., 2017). The common mechanism of antidepressants in the treatment of depression is to enhance the synaptic plasticity of neurons (Mitra et al., 2006; Jayatissa et al., 2008). Synaptic plasticity changes are closely bound up with the expression of synaptic proteins. Postsynaptic PSD 95 and presynaptic SYN 1 are the main protein markers of synapses. PSD 95, one of the postsynaptic dense protein family and a key marker of the postsynaptic membrane, regulates synaptic transmission and synaptic function (Sheng and Hoogenraad, 2007). SYN 1, a specifically labeled protein of synaptic vesicles, reflects the synaptic number, density, and distribution (Rajappa et al., 2016). PSD 95 and SYN 1 play a vital role in promoting signaling and synaptic plasticity. Damaged hippocampal neurons and disturbed synaptic plasticity have been found in CUMS model rats, and depression-like behaviors can be significantly improved after treatment with some antidepressants (Feyissa et al., 2009; Zhang et al., 2018; Lu et al., 2021). Our study findings indicated that PSD 95 and SYN 1 protein levels were significantly reduced in the model group. Both were remarkably elevated after honokiol treatment, confirming that honokiol can improve synaptic plasticity in CUMS depression rat models. The antidepressant mechanism of honokiol is illustrated in Figure 14.

Molecular docking is an important technology for predicting affinity through the interaction between ligand and receptor to realize structure-based drug design (Xiao et al., 2022). The results showed that all core targets had good docking activity with honokiol (binding energy < −5.0 kcal/mol), further confirming that honokiol can play a therapeutic role in depression by modulating the HIF-1α-VEGF pathway.

Currently, significant differences have been observed in testing protein expression on the basis of *n* = 3 per group. The western blot results presented were treated as observations due to the number of animals tested. In future studies, samples size should be increased to further confirm the reliability of the results.

## Conclusion

Our results suggest that honokiol has an antidepressant effect in CUMS model rats. The observed beneficial effects may be attributed to the activation of the HIF-1α-VEGF signaling pathway, VEGFR-2-mediated PI3K/AKT/mTOR signaling pathway, and increased expression of the synaptic plasticity-related proteins, SYN 1 and PSD 95. The results also show that *in vitro*, honokiol enhances synaptic plasticity in PC12 cells by activating the HIF-1α-VEGF pathway.

## Data Availability

The original contributions presented in the study are included in the article/Supplementary Material, further inquiries can be directed to the corresponding authors.

## Abbreviations

2-ME, 2-methoxyestradiol; CNS, central nervous system; CUMS, chronic unpredictable mild stress; HIF-1α, hypoxia-inducible factor-1α; HP, hypoxic preconditioning; HPA, hypothalamus-pituitary-adrenal; HREs, hypoxia response elements; IH, intermittent hypoxia; MAOIs, monoamine oxidase inhibitors; OFT, open field tests; PSD 95, postsynaptic density protein 95; SPT, sucrose preference tests; SSRIs, selective serotonin reuptake inhibitors; SYN 1, synapsin 1; TCAs, tricyclic antidepressants; VEGF, vascular endothelial growth factor; VEGFR-2, vascular endothelial growth factor receptor 2.
