# Supplementary figures and images for: Honokiol improves depression-like behaviors in rats by HIF-1α- VEGF signaling pathway activation
Source: Front Pharmacol. 2022 Aug 25;13:968124. doi: 10.3389/fphar.2022.968124 (PMC9453876; doi:10.3389/fphar.2022.968124)

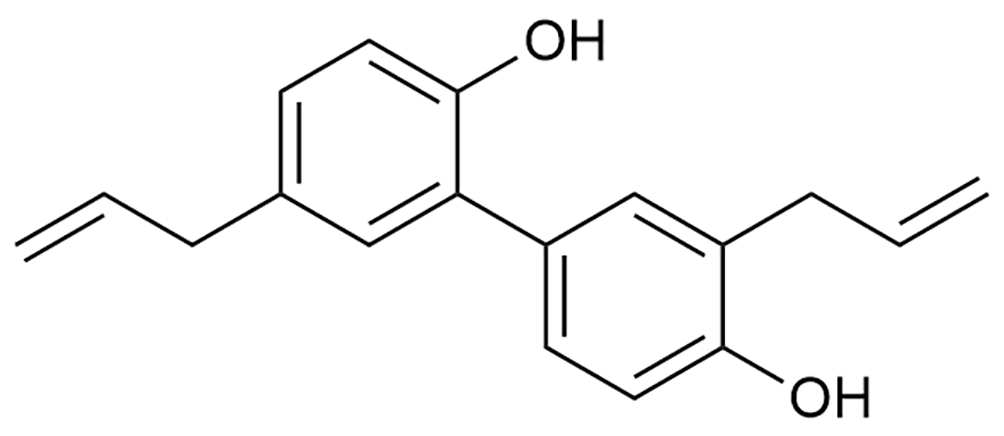

Supplement: Supplementary file 1 [file DataSheet1.ZIP › Supplementary Material Presentation/Figure 1.tif]

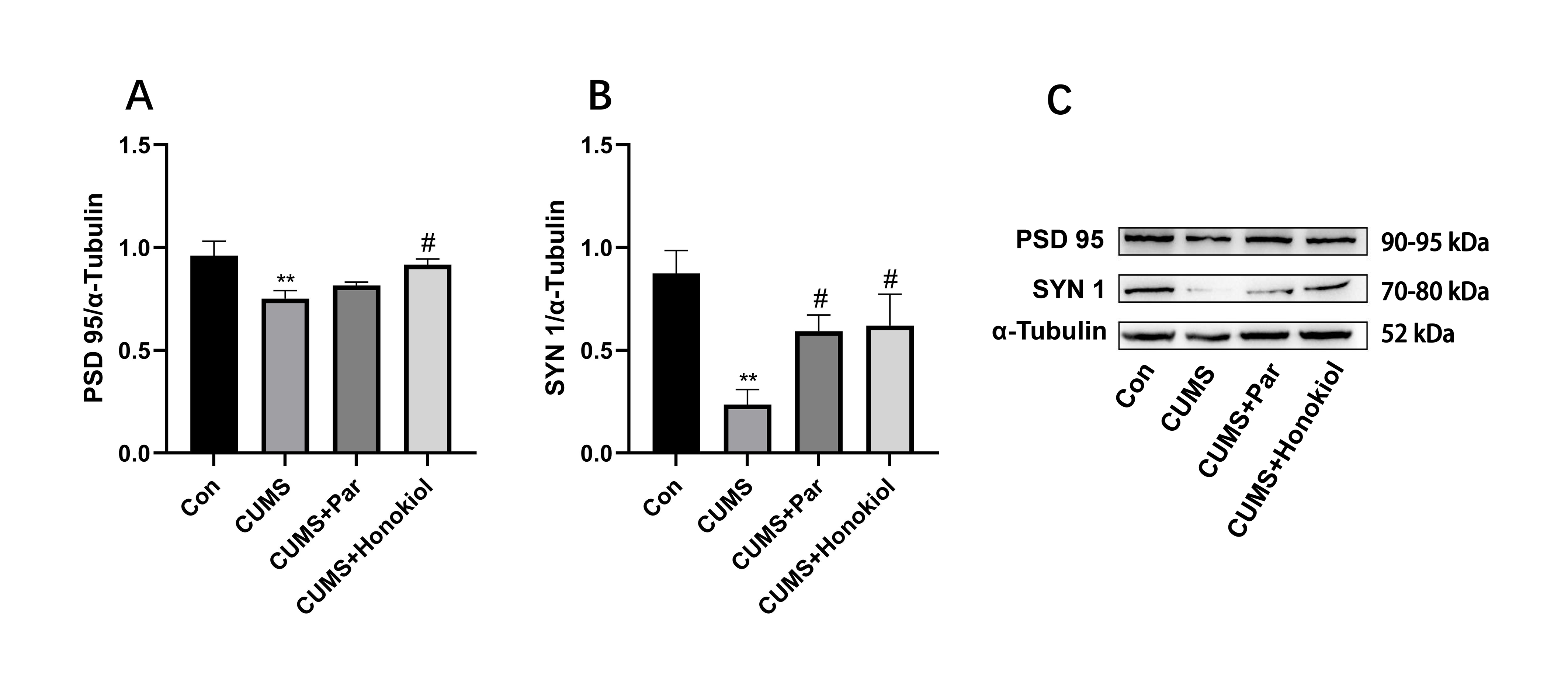

Supplement: Supplementary file 1 [file DataSheet1.ZIP › Supplementary Material Presentation/Figure 10.tif]

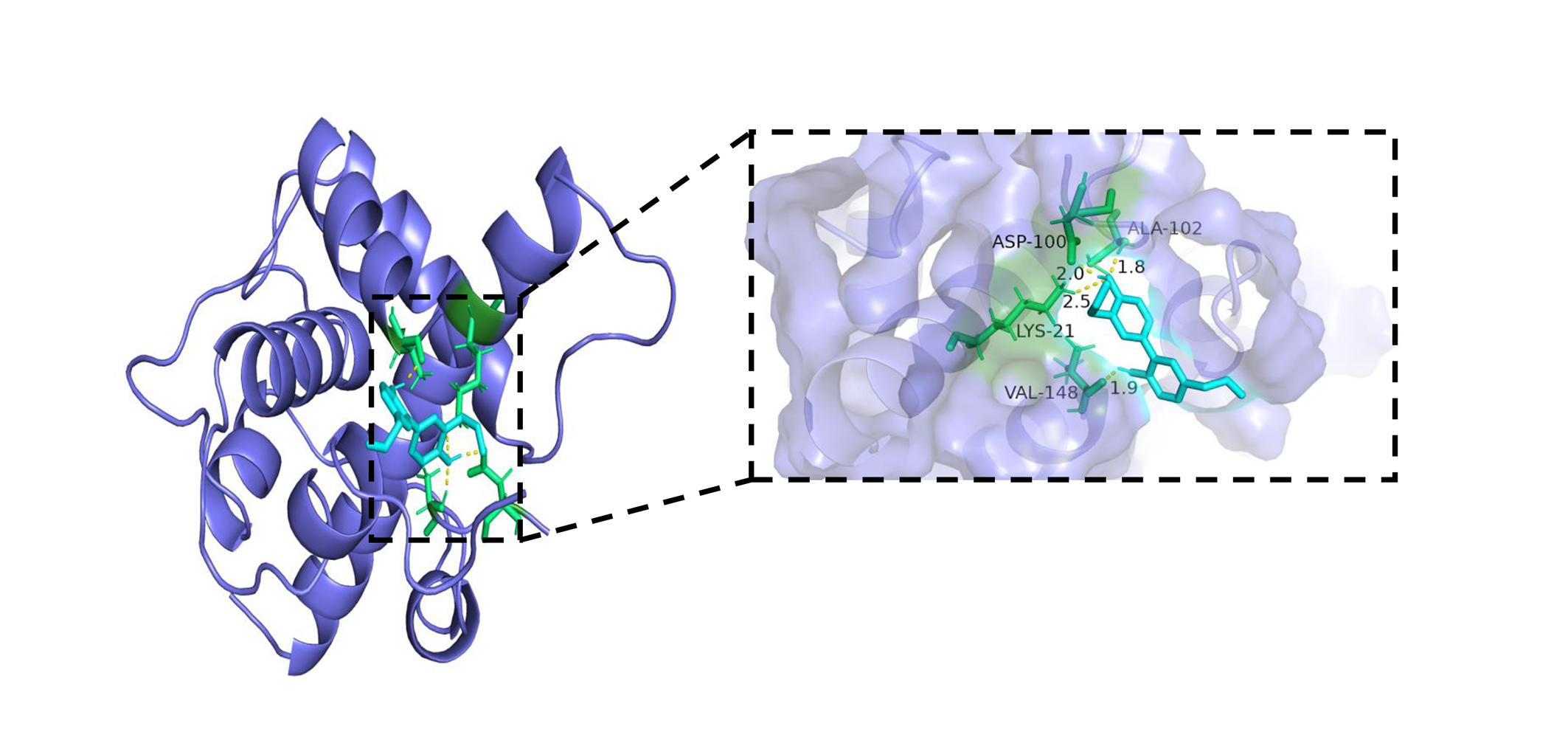

Supplement: Supplementary file 1 [file DataSheet1.ZIP › Supplementary Material Presentation/Figure 11.tif]

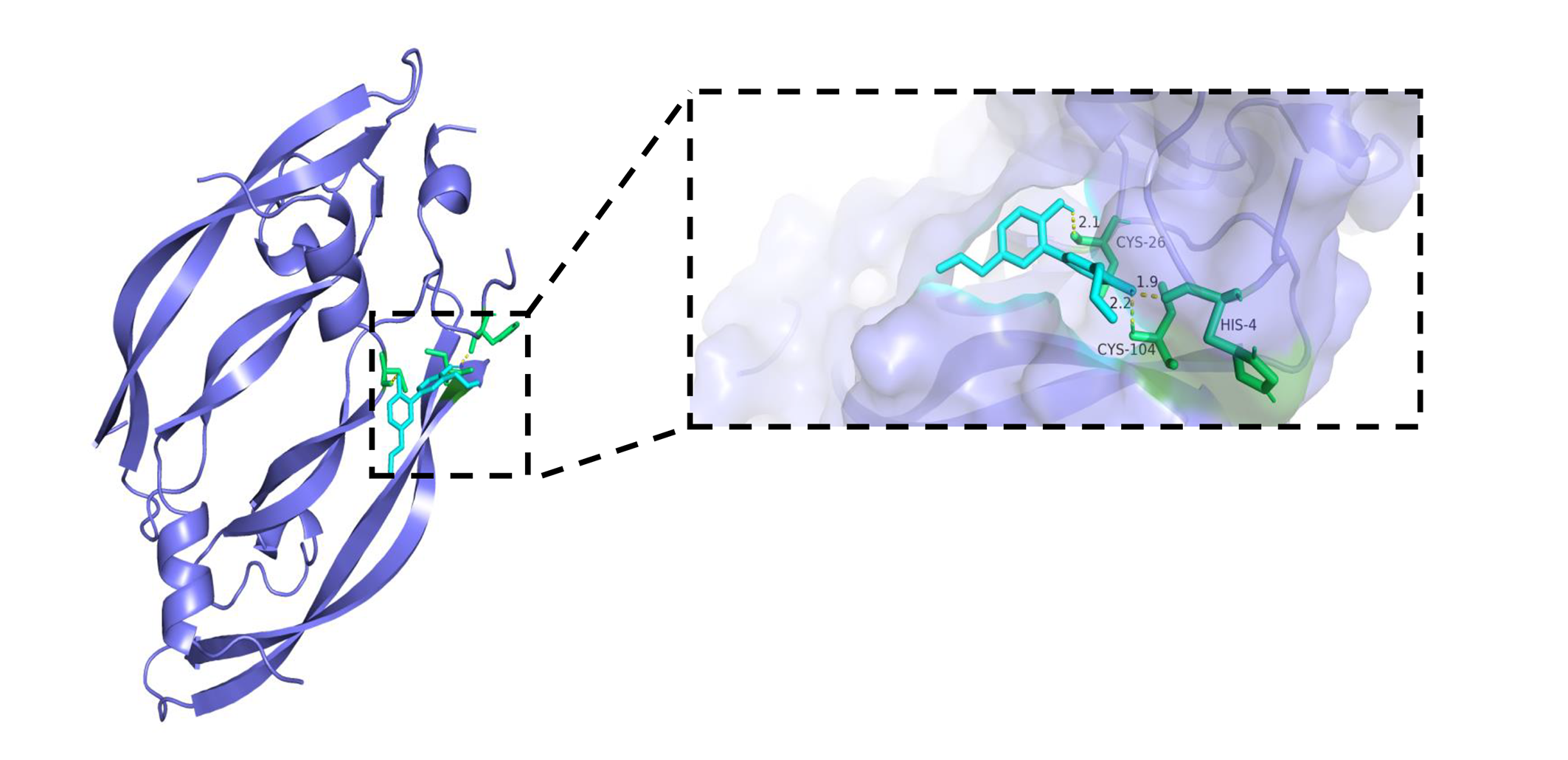

Supplement: Supplementary file 1 [file DataSheet1.ZIP › Supplementary Material Presentation/Figure 12.tif]

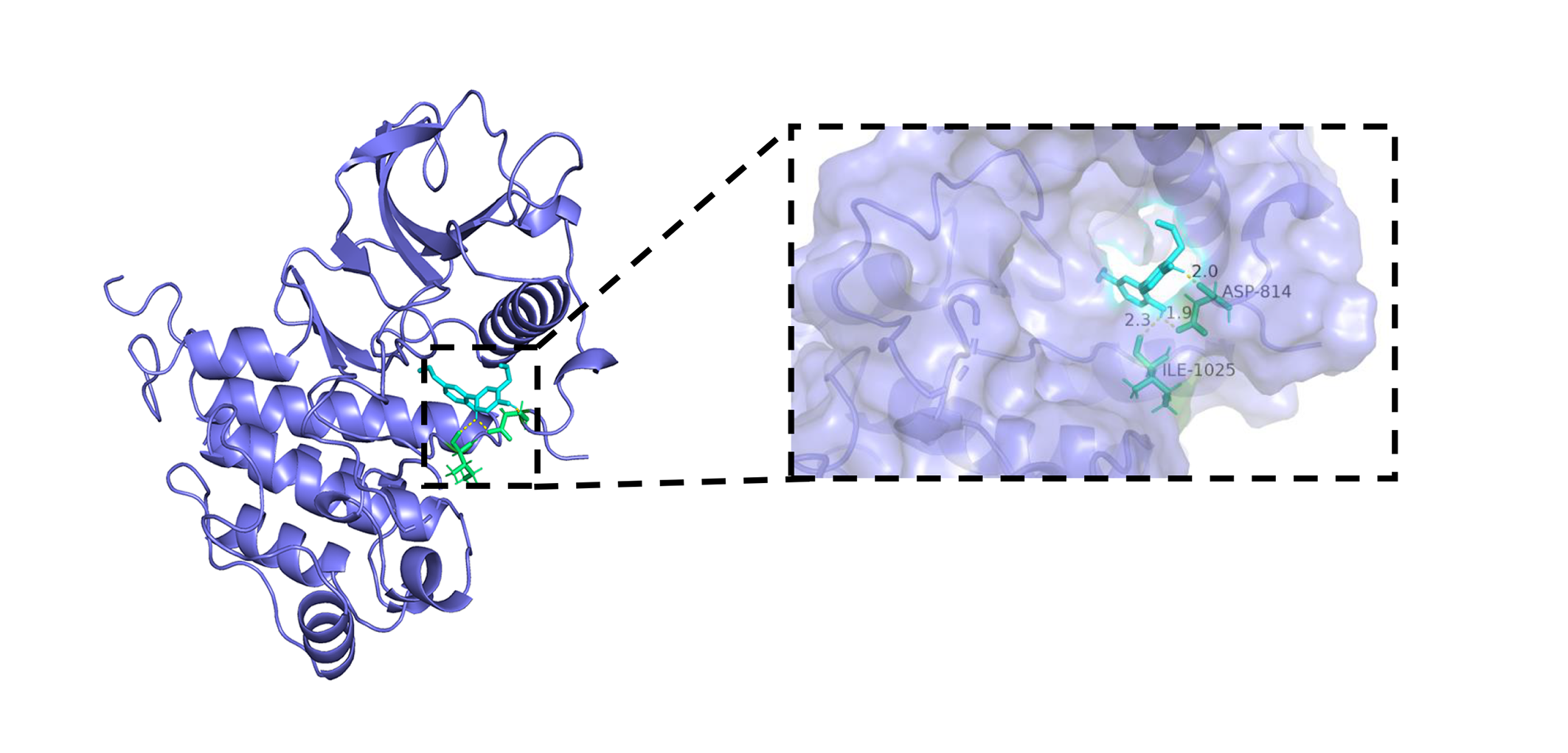

Supplement: Supplementary file 1 [file DataSheet1.ZIP › Supplementary Material Presentation/Figure 13.tif]

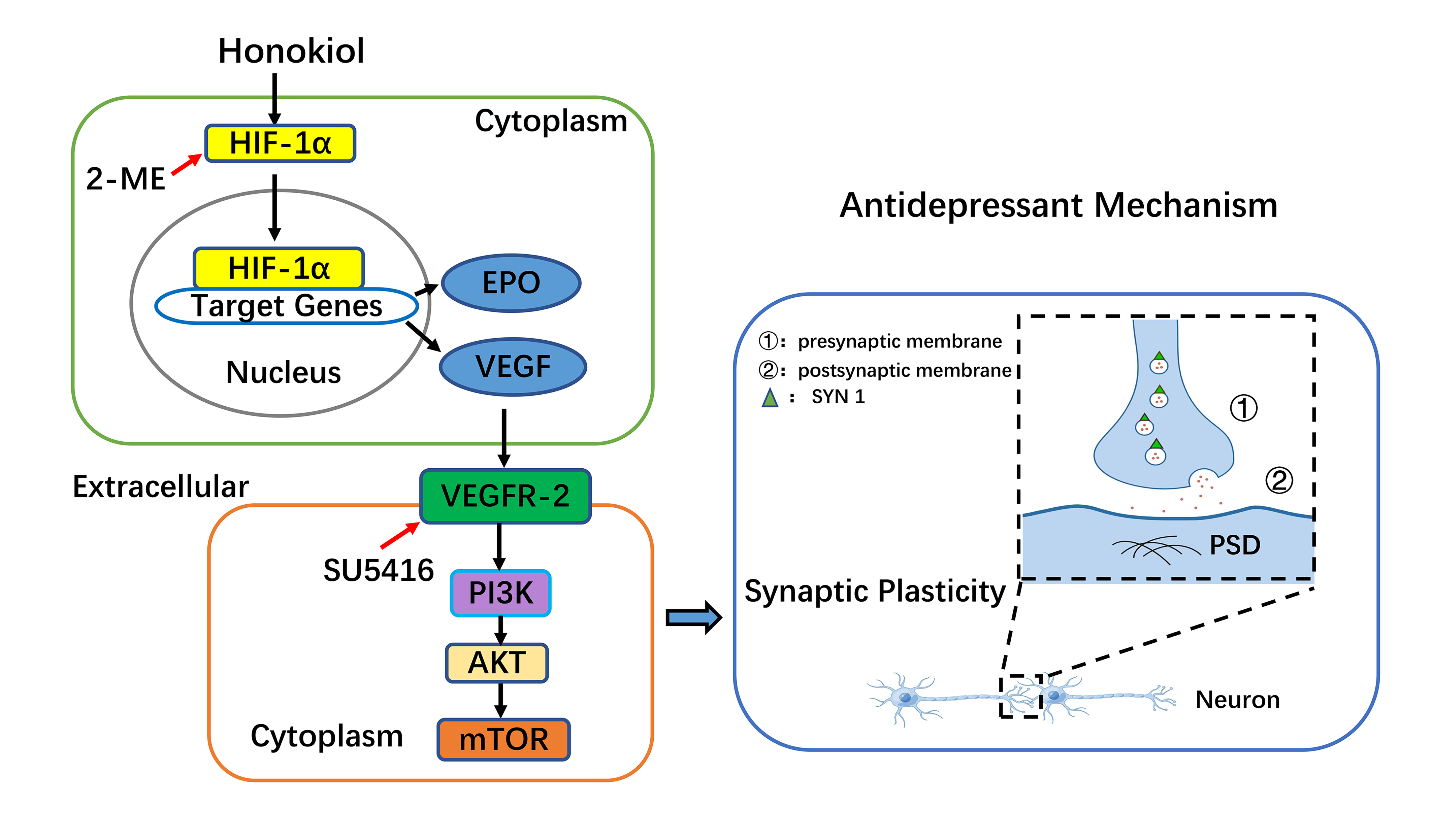

Supplement: Supplementary file 1 [file DataSheet1.ZIP › Supplementary Material Presentation/Figure 14.tif]

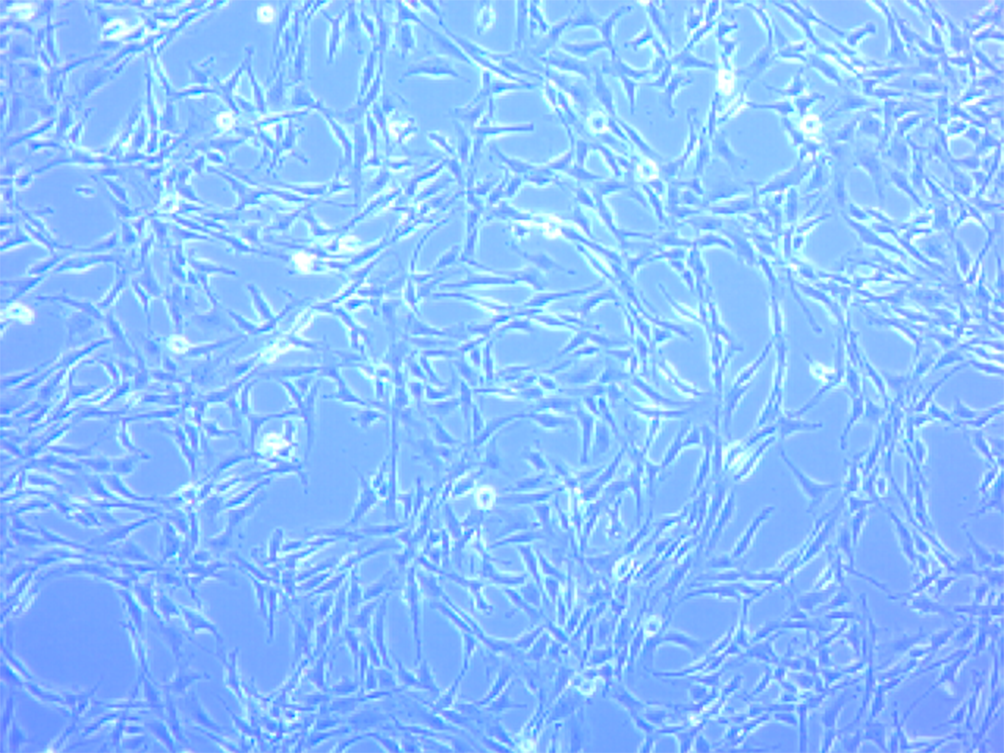

Supplement: Supplementary file 1 [file DataSheet1.ZIP › Supplementary Material Presentation/Figure 2.tif]

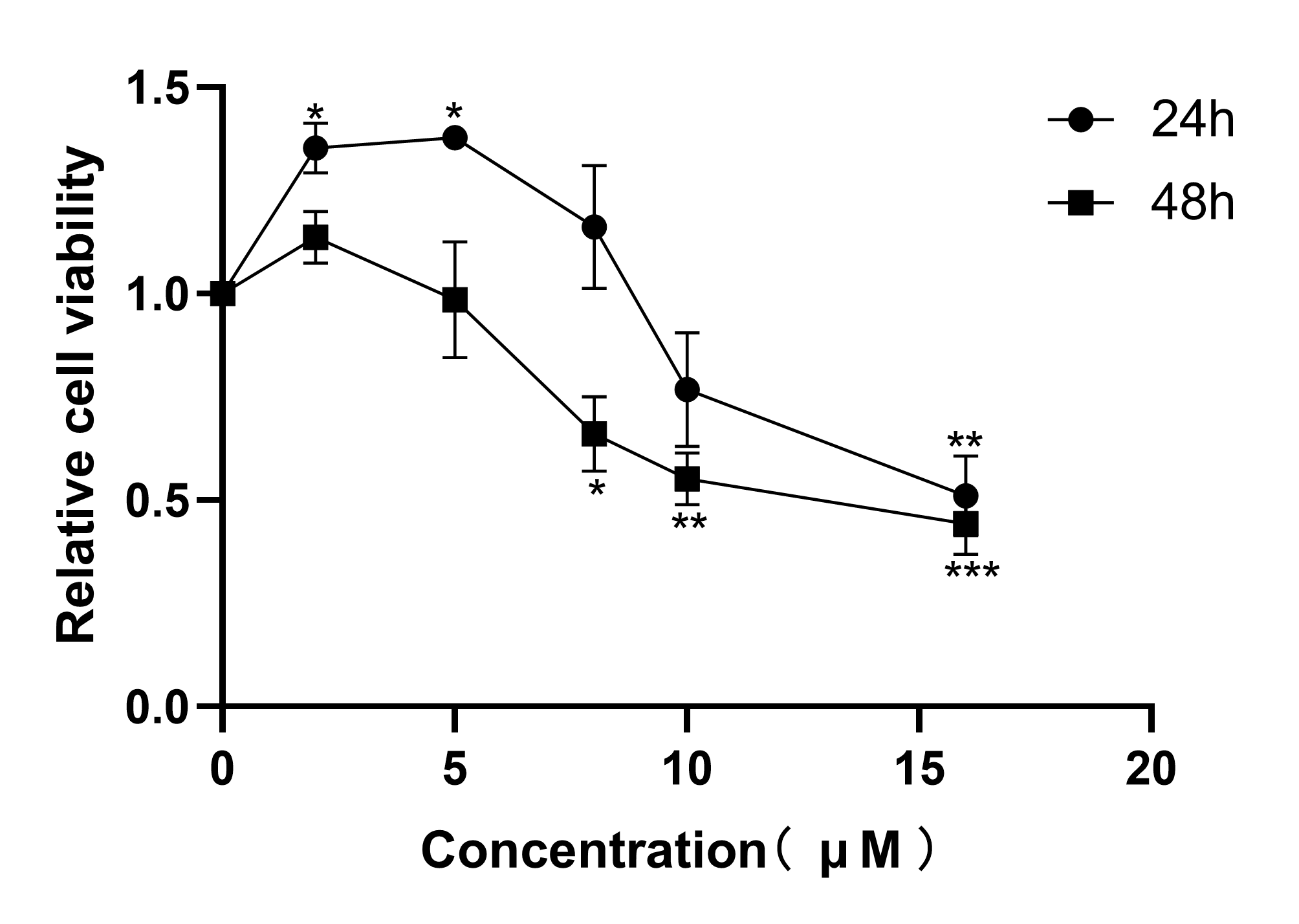

Supplement: Supplementary file 1 [file DataSheet1.ZIP › Supplementary Material Presentation/Figure 3.tif]

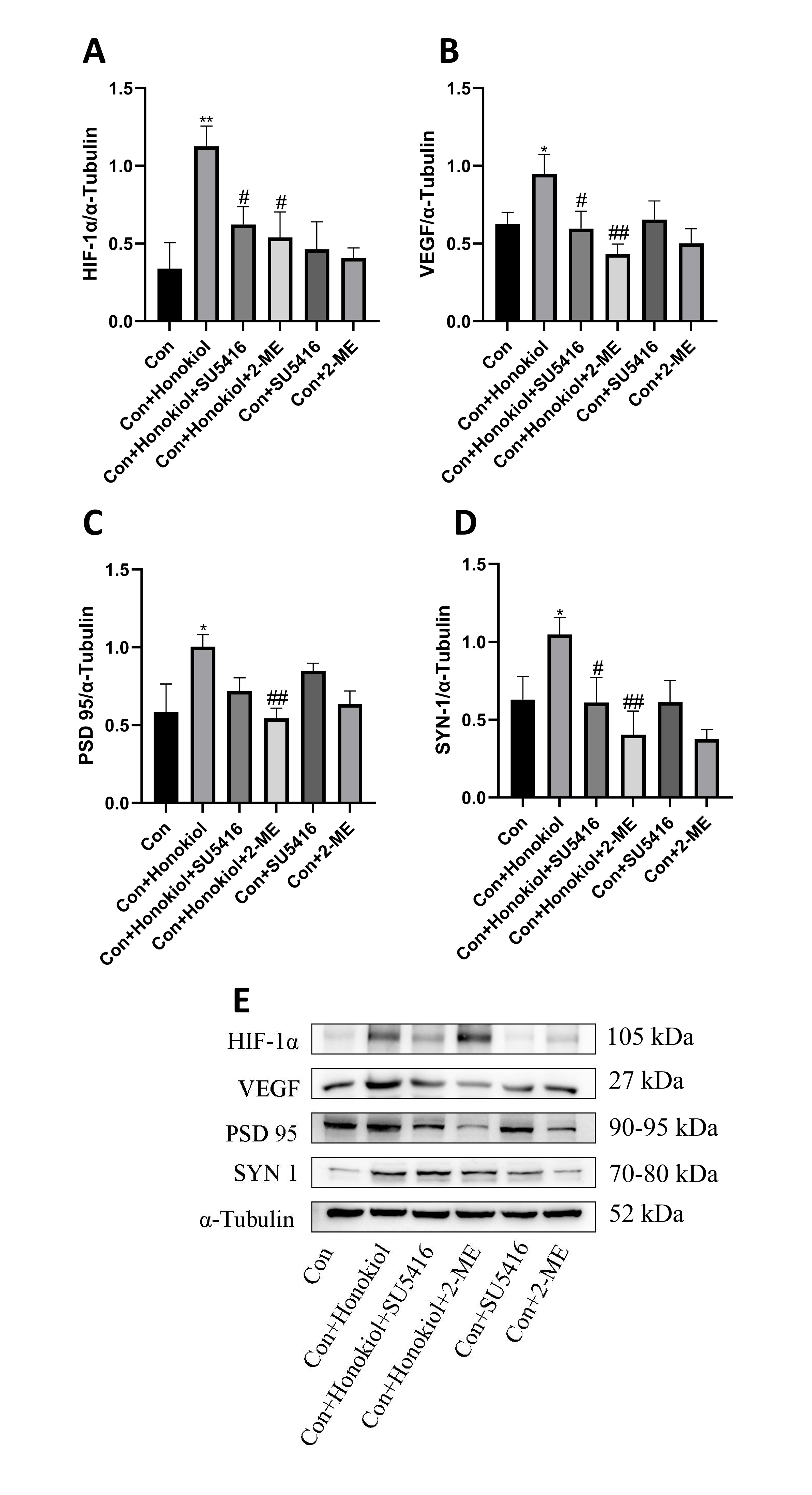

Supplement: Supplementary file 1 [file DataSheet1.ZIP › Supplementary Material Presentation/Figure 4.tif]

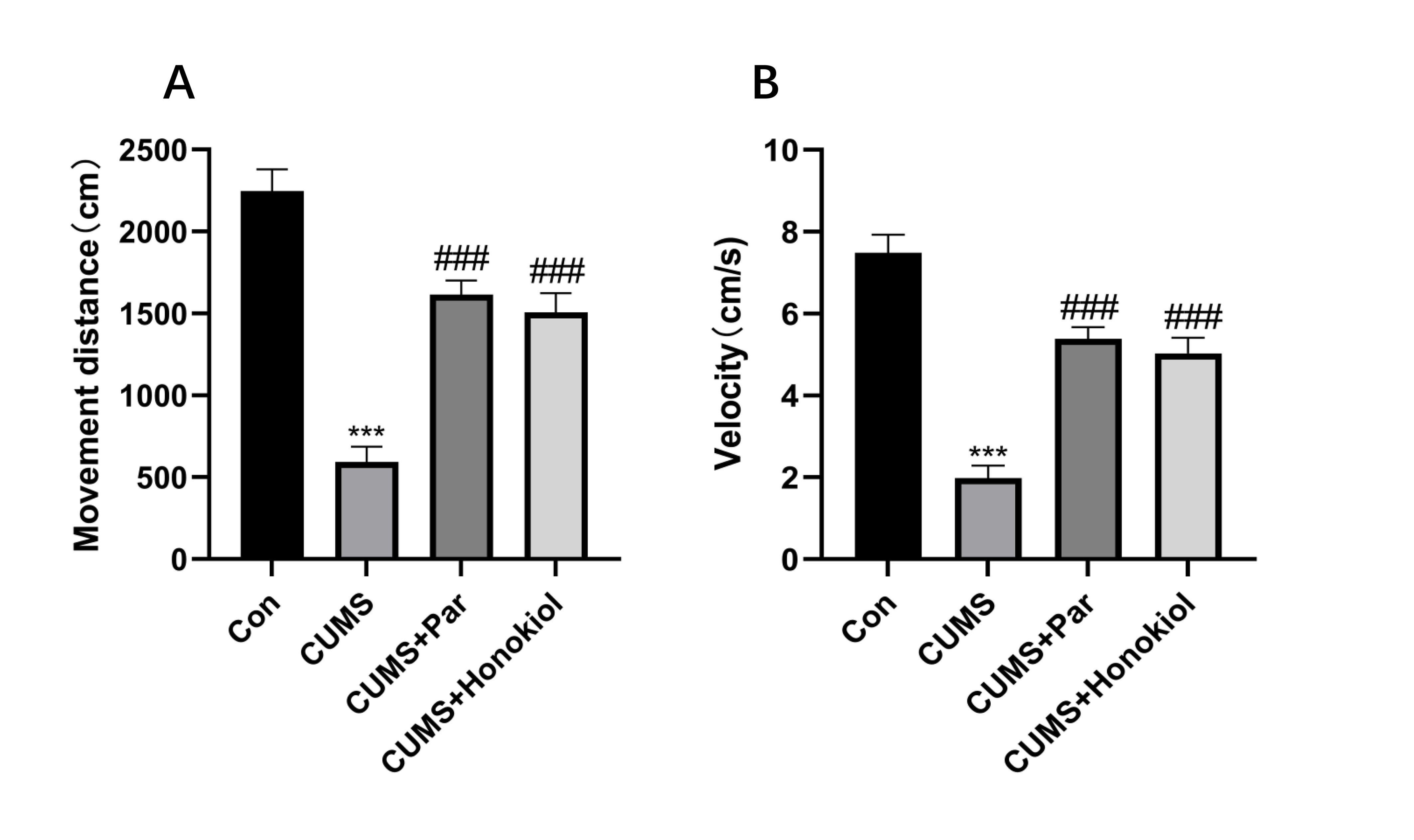

Supplement: Supplementary file 1 [file DataSheet1.ZIP › Supplementary Material Presentation/Figure 5.tif]

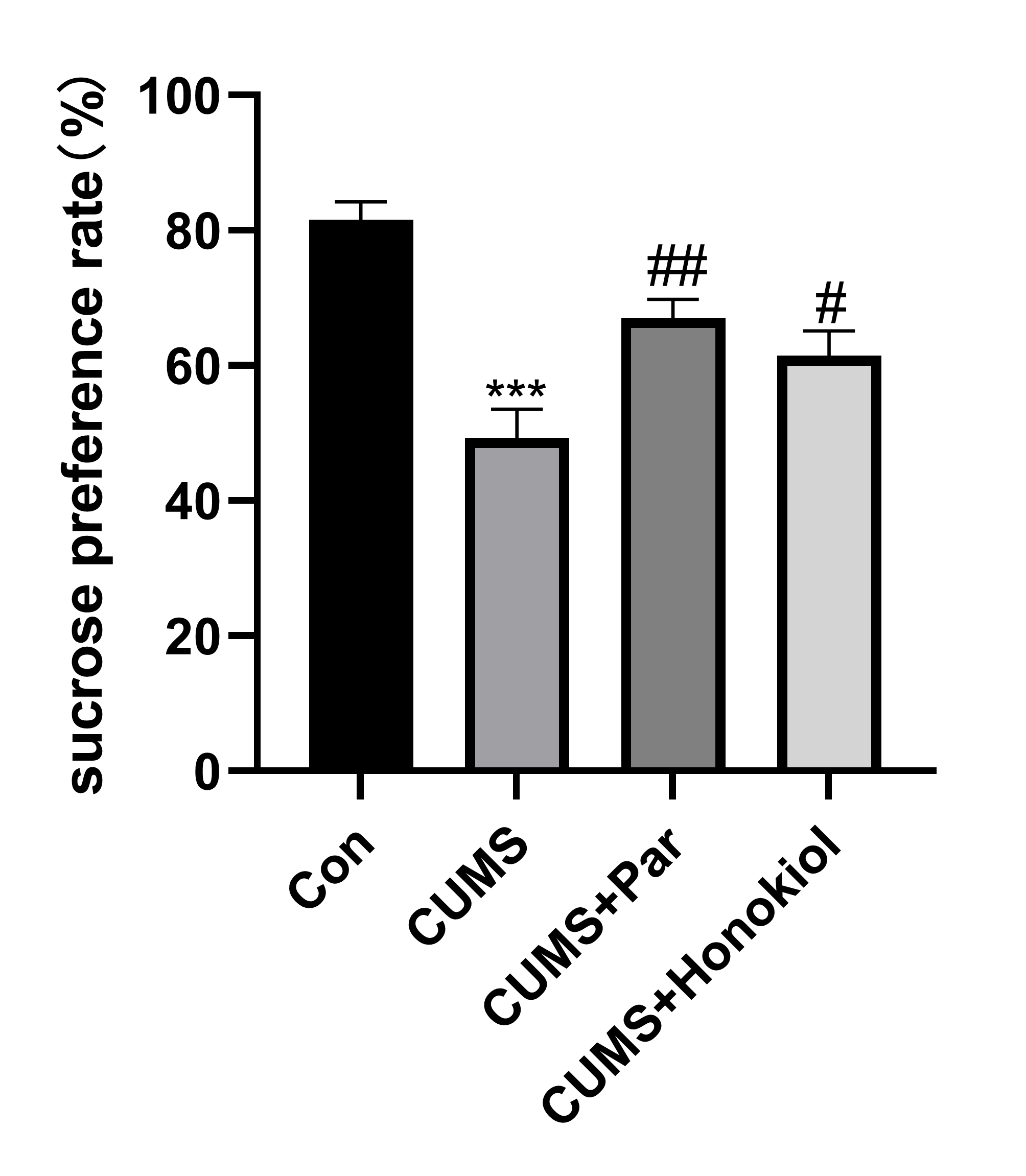

Supplement: Supplementary file 1 [file DataSheet1.ZIP › Supplementary Material Presentation/Figure 6.tif]

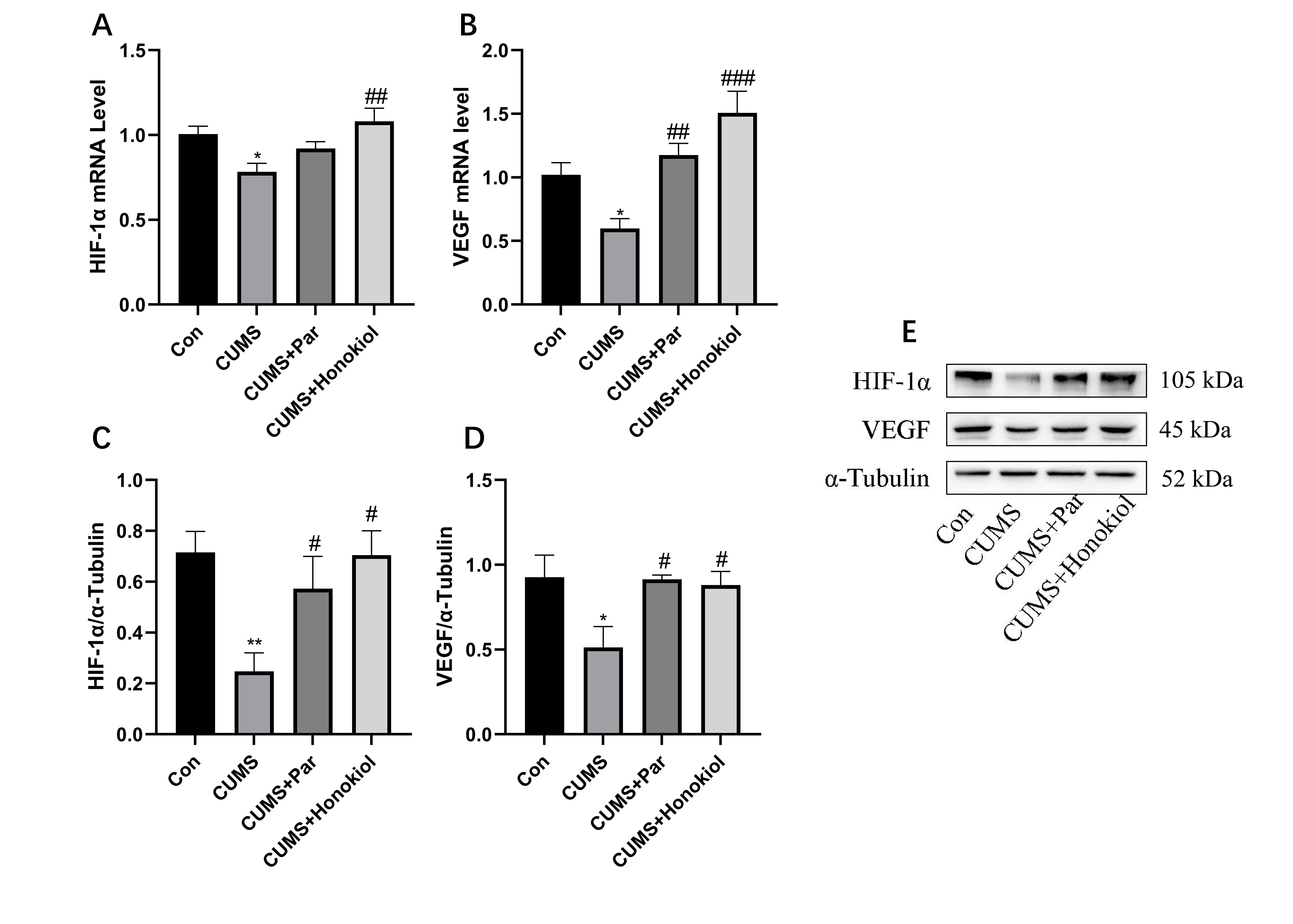

Supplement: Supplementary file 1 [file DataSheet1.ZIP › Supplementary Material Presentation/Figure 7.tif]

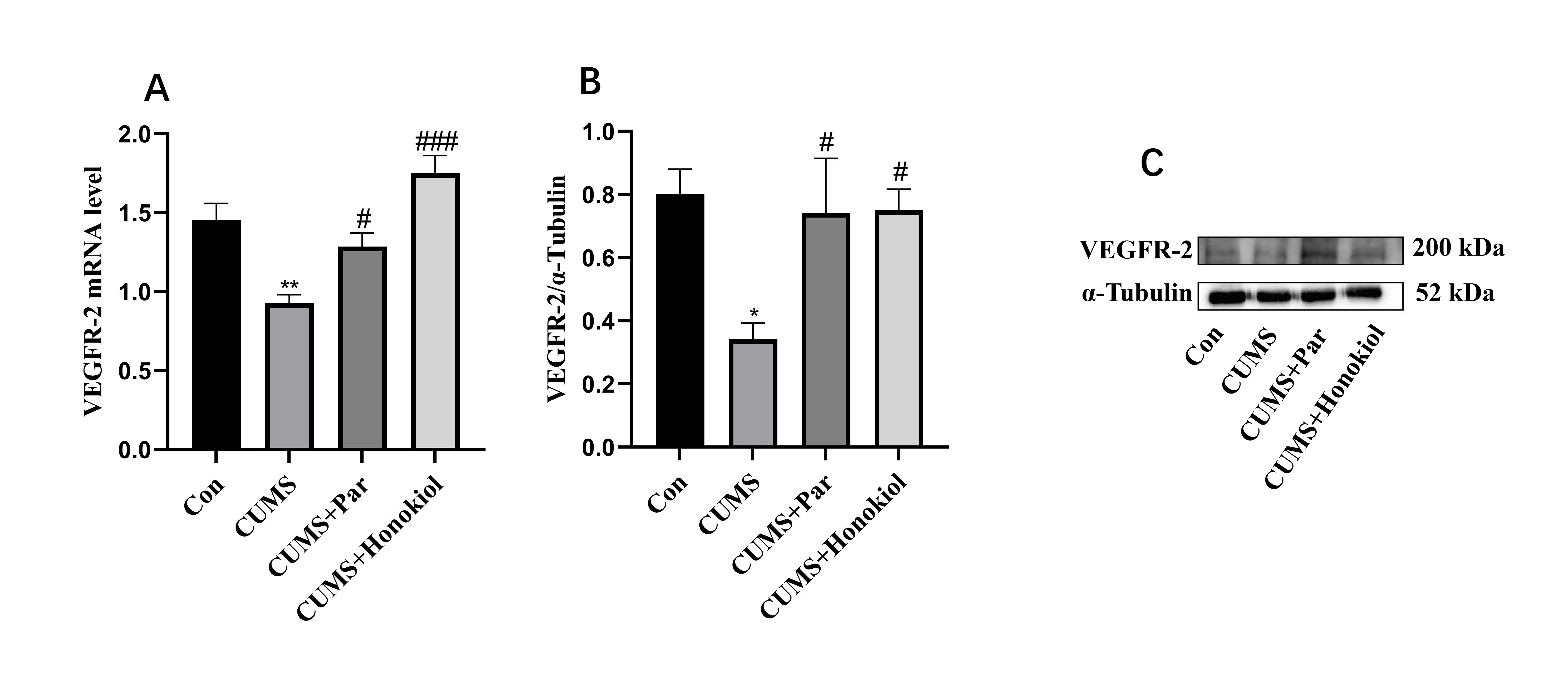

Supplement: Supplementary file 1 [file DataSheet1.ZIP › Supplementary Material Presentation/Figure 8.tif]

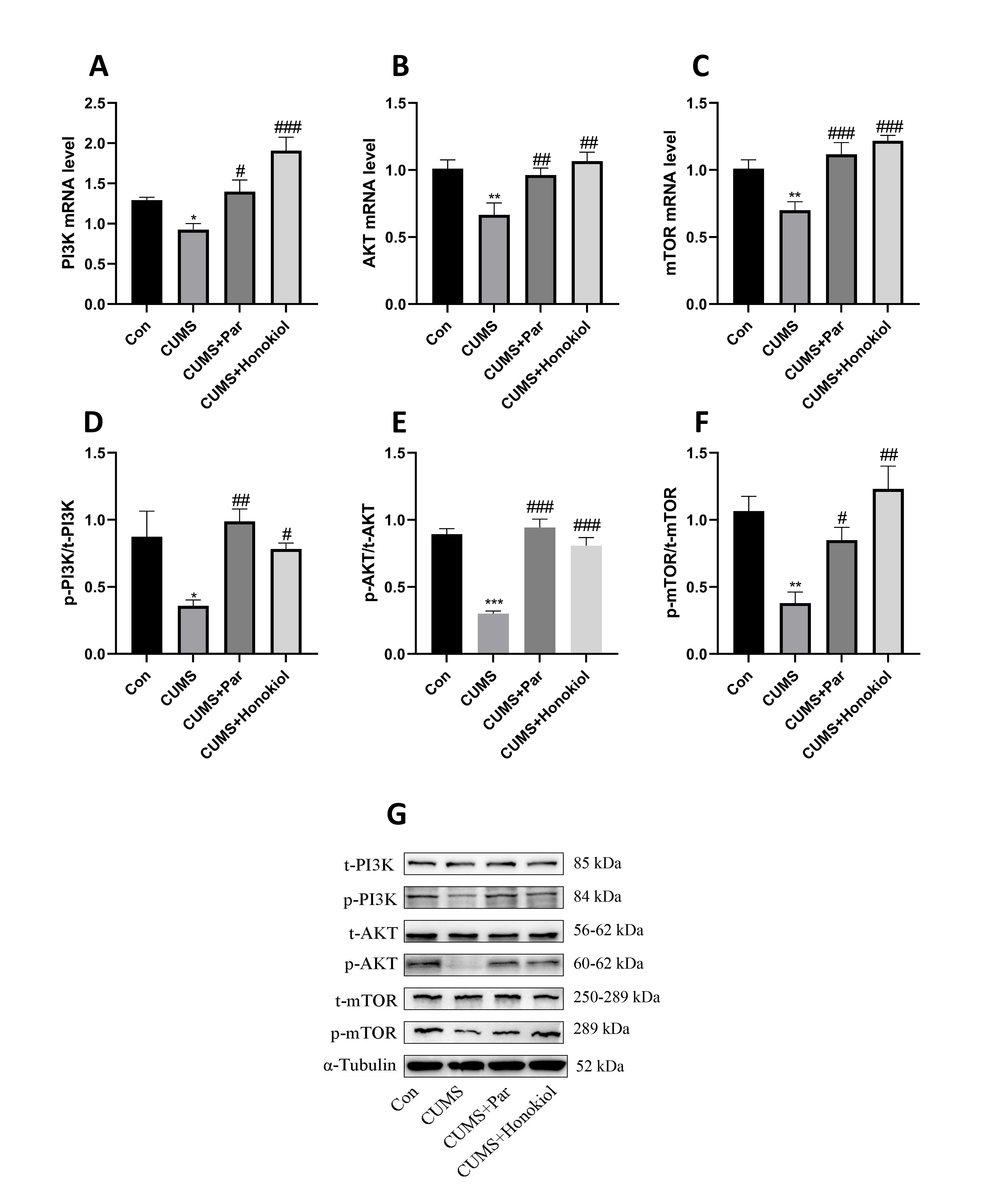

Supplement: Supplementary file 1 [file DataSheet1.ZIP › Supplementary Material Presentation/Figure 9.tif]
